# Inference of Multiple Mergers while Dating a Pathogen Phylogeny

**DOI:** 10.1093/sysbio/syaf003

**Published:** 2025-01-18

**Authors:** David Helekal, Jere Koskela, Xavier Didelot

**Affiliations:** Centre for Doctoral Training in Mathematics for Real-World Systems, University of Warwick, Coventry CV47AL, UK; School of Mathematics, Statistics and Physics, Newcastle University, Newcastle NE17RU, UK; School of Life Sciences and Department of Statistics, University of Warwick, Coventry CV47AL, UK

**Keywords:** Coalescent model, multiple mergers, pathogen phylogenetics, phylogenetic dating

## Abstract

The vast majority of pathogen phylogenetic studies do not consider the possibility of multiple merger events being present, where a single node of the tree leads to more than 2 descendent branches. These events are however likely to occur when studying a relatively small population or if there is high variability in the reproductive chances. Here we consider the problem of detecting the presence of multiple mergers in the context of dating a phylogeny, that is determining the date of each of the nodes. We use the Lambda-coalescent theory as a modeling framework and show how Bayesian inference can be efficiently performed using a Billera-Holmes-Vogtmann space embedding and a customized Markov Chain Monte Carlo sampling scheme. We applied this new analysis methodology to a large number of simulated data sets to show that it is possible to infer if and when multiple merger events occurred, and that the phylogenetic dating is improved as a result of taking this information into account. We also analyzed real data sets of *Vibrio cholerae* and *Mycobacterium tuberculosis* to demonstrate the relevance of our approach to real pathogen evolutionary epidemiology. We have implemented our new methodology in a R package, which is freely available at https://github.com/dhelekal/MMCTime.

Dated phylogenies have risen to prominence in many research areas of the life sciences, from the study of evolutionary histories of higher organisms, genomic epidemiology of infectious disease, through to understanding diversity of microbial organisms. Most existing approaches to reconstructing and analyzing dated phylogenies are restricted to binary trees, where each internal node has exactly 2 descendent branches. Indeed, Kingman’s coalescent (Kingman  1982) on a continuous real time scale (Drummond et al.  2002) is a popular framework for modeling dated phylogenies of measurably evolving populations (Drummond et al.  2003; Biek et al.  2015), and in this model only 2 lineages may merge into the same ancestor at once. However, this model is only applicable if both the sample size and typical family sizes are small in comparison to the effective population size, which can be orders of magnitude smaller than the census population size for example due to heterogeneity of the reproduction success (Charlesworth  2009).

In contrast with the standard Kingman’s coalescent, Lambda-coalescent models, also known as multiple merger coalescents, can be used to describe dated phylogenies where more than 2 lineages may coalesce into the same ancestor at once (Pitman 1999; Sagitov  1999; Donnelly and Kurtz  1999). Multiple merger events can be the result of various biological phenomena of interest, such as populations undergoing rapid adaptation (Neher and Hallatschek  2013; Desai et al.  2013), superspreading (Hoscheit and Pybus  2019; Lemieux et al.  2021; Gómez-Carballa et al.  2021), or some other form of sweepstakes reproduction (Menardo et al.  2021; Árnason et al.  2023). In particular, the Beta-coalescent (Schweinsberg  2003) is a specific type of Lambda-coalescent that has been used to explain the shallow genealogies observed in cod (Birkner et al.  2013), to study pathogen superspreading (Hoscheit and Pybus  2019) and to characterize *Mycobacterium tuberculosis* outbreak genealogies (Menardo et al.  2021).

Let us take as our starting point an unrooted, undated tree produced by a maximum likelihood tree reconstruction software such as RAxML (Stamatakis  2014), IQ-TREE (Minh et al.  2020), or PhyML (Guindon et al.  2010). Such a tree may contain polytomies, where a node leads to more than 2 branches. This can be either because of a multiple merger event, or because of at least 1 branch covering a short interval of time, so that no substitution occurred as expected under any molecular clock model (Bromham and Penny  2003). Multiple heuristic approaches have been developed for either breaking up or collapsing polytomies in undated phylogenies (Kuhn et al.  2011; Lin et al.  2011; Lewis et al.  2005). Here instead we use a Lambda-coalescent framework to infer which polytomies are caused by multiple merger events and which are caused by a lack of phylogenetic signal. We do so in the context of dating the tree, which means to use it as well as the dates of the leaves in order to produce a dated phylogeny (To et al.  2016; Volz and Frost  2017; Didelot et al.  2018). The dated phylogeny can then be used for a broad range of epidemiological investigations (Didelot and Parkhill  2022). To reconstruct this dated phylogeny, we must infer the root position, ancestral node times, as well as parameters associated with the clock and genealogical models. We must distinguish which polytomies are consistent with multiple mergers, and which are better explained by quick binary branching and therefore should be resolved. In the latter case, we must also estimate the branching order within the polytomies returned by the maximum likelihood estimation software. This is important as the branching order within the polytomies is random and likely inconsistent with the temporal structure of the tree, as previously noted (Sagulenko et al.  2018).

To achieve these aims several issues must be addressed. We need to choose a set of prior models for the latent genealogies which take into account biological realism and statistical tractability. We focus on the Beta-coalescent (Schweinsberg  2003) and an extension of it described by Eldon and Stephan (2024) in which the Beta-coalescent and Kingman’s coalescent are mixed together, combining low-variance family size reproduction with occasional high-variance sweepstakes. We also need to specify a molecular clock model to establish the relationship between dated and undated phylogenies, and for this we use the Additive Relaxed Clock (ARC) model (Didelot et al.  2021). Next, we need to specify a computational scheme for representing multiple merger trees where a single node may have an arbitrary number of descendants. This representation needs to enable efficient computation of likelihoods and to be statistically efficient. To this end we use the Billera-Holmes-Vogtmann (BHV) space (Billera et al.  2001) augmented with a spike-and-slab construction (George and McCulloch  1993). Finally, we design a Markov Chain Monte-Carlo (MCMC) sampling scheme targeting the posterior in order to perform Bayesian inference.

## METHODS

### Lambda-Coalescent

Lambda-coalescents are a class of stochastic genealogical processes (Pitman 1999; Sagitov  1999; Donnelly and Kurtz  1999) that generalize the popular Kingman’s coalescent (Kingman  1982) to a setting where more than 2 lineages may merge into the same parent, that is, they permit multiple mergers. These processes commonly describe genealogies arising from various forwards in time models in population genetics, typically in scenarios where there is a high variability in the number of surviving offspring, or when selection and recombination are taken into account (Berestycki  2009; Tellier and Lemaire  2014). Examples of such scenarios include heavy-tailed offspring distributions (Schweinsberg  2003; Matuszewski et al.  2018), recurrent selective sweeps in presence of recombination (Durrett and Schweinsberg  2005), rapidly adapting populations (Neher and Hallatschek  2013; Desai et al.  2013), as well as strong purifying selection (Cvijovic et al.  2018).

Lambda-coalescents are uniquely specified by a finite measure Λ on [0,1] that governs the merger sizes of the process. Intuitively, one can think of this relationship as sampling a probability p∼Λ proportional to the density of Λ, selecting with probability p each of the lineages currently in the process, and merging all selected lineages. The instantaneous block merger rates $\lambda_{b,k}$, that is the rate at which every k≥2 lineages merges into 1 parent when there are b≥2 lineages in total, are then given by


$$\begin{array}{ccc} \lambda_{b,k}=\int_{0}^{1}p^{k-2}{\left(1-p\right)}^{b-k}\;\text{Λ}(dp). & & \end{array}$$ (1)

The factor of $p^{-2}$ arises from the fact that at least 2 lineages must participate in order for a merger to happen. When $\text{Λ}=\delta_{0}$, that is a point mass at 0, we can see that


$$\begin{array}{ccc} \lambda_{b,k}=\{\begin{array}{c} 1,\;if\;k=2\;\\ 0,\;\text{otherwise}\; \end{array} & & \end{array}$$ (2)

In other words the resulting Lambda-coalescent is Kingman’s coalescent in which only 2 lineages may merge.

### Beta-Coalescent

The approach presented will mostly focus on the Beta-coalescent and an extension of it. The rationale for this is 2-fold. First, the Beta-coalescent is relatively well studied, admits the frequently used Kingman’s coalescent as a special case, and the instantaneous block merger rates are available in closed form. Second, it arises from models in which the variance of the offspring distribution can be very high, and has been considered in the context of pathogen populations before, see, for example (Hoscheit and Pybus  2019). As the name suggests, in the case of the Beta-coalescent the measure Λ is simply the Beta distribution. Usually in the context of Lambda-coalescents, the Beta distribution is parameterized as (Schweinsberg  2003)


$$\begin{array}{ccc} \text{Λ}=Beta\;(2-\alpha ,\alpha )\;\alpha \in [1,2] & & \end{array}$$ (3)

The reason for this is a connection to a model of populations with skewed offspring distributions in which α governs the tail behavior of the offspring distribution. The forwards-in-time model in the derivation of (Schweinsberg  2003) follows dynamics of a supercritical Galton–Watson process where in successive non-overlapping generations each of the N individuals produces $\nu_{i}$ offspring i.i.d. according to a distribution with the tail index $k^{-\alpha }$. This implies that the offspring distribution has infinite variance if 1<α<2 and infinite mean if α=1. Offsprings are then randomly killed in order to keep the population size constant and equal to N. Schweinsberg (2003) showed that the genealogies arising for this process converge to Kingman’s coalescent for values of α≥2 and to the Beta-coalescent parameterized as in Equation 3 for values of α∈(2,1], under suitable time-rescalings and as N→∞. In plain words if the distribution of the number of offspring produced by any of the individuals is sufficiently skewed, and this situation arises frequently enough, every once in a while an individual may get lucky and produce enough offspring to replace a non-negligible fraction of the population. Therefore, the probability that multiple individuals find the same ancestor at once does not vanish in the large population size limit. The parameter α relates to how skewed the offspring distribution is. The limiting case of α=1 corresponds to the Bolthausen-Sznitman coalescent (Bertoin and Le Gall  2000), which has been shown to arise in scenarios corresponding to rapid adaptation and clonal interference (Desai et al.  2013; Neher and Hallatschek  2013; Schweinsberg  2017). On the other hand, in the limiting case of α=2, the Beta distribution collapses into an atom at 0 and thus the resulting coalescent is the Kingman’s coalescent (Schweinsberg  2003).

There are several limitations of the Beta-coalescent. First amongst these is the assumption that every individual may produce a large number of offspring in every generation. Often it is easier to imagine that such large reproductive events may only occur if correct circumstances are met. Related to this is the second limitation. The Beta-coalescent has a time scale of $N^{1-\alpha }$ if α∈(1,2] and $\log{\left(N\right)}^{-1}$ if α=1. For many populations, this implies mutation rates or population sizes orders of magnitude higher than what would be biologically realistic. This is especially the case if α is close to 1 as previously noted (Eldon and Stephan  2024). This may however not be an issue for moderately large values of α in the case of within-host evolution where the population sizes are going to be very large.

### Extended Beta-Coalescent

We now introduce an extension of the Beta-coalescent which we will refer to as the extended Beta-coalescent. This modification is a mixture of the Beta-coalescent and Kingman’s coalescent. This is achieved by defining the measure Λ characterizing this process as


$$\begin{array}{ccc} \text{Λ}=\delta_{0}+cBeta(2-\alpha ,\alpha )\;c\in [0,\infty ),\;\alpha \in [1,2] & & \end{array}$$ (4)

The reasons for introducing this are 2-fold. For one, it will provide us with a convenient example of a Lambda-coalescent of a form shared by for example the Durrett–Schweinsberg coalescent (Durrett and Schweinsberg  2005) arising from selective sweeps. The second reason is based on the modifications to random sweep-stakes reproduction presented in (Eldon and Stephan  2024). They considered a modification to the construction in (Schweinsberg  2003) to address the problematic assumption of very frequent large family sizes. In this modification, in each generation a coin with probability ϵ is flipped. On success with probability ϵ each individual produces offspring according to an offspring distribution with a “small” α∈[1,2) and with probability 1−ϵ according to an offspring distribution α≥2. For a suitable choice of $\epsilon =\epsilon_{N}\to 0$ as N→∞, the resulting coalescent process is exactly the Lambda-coalescent specified by Equation 4.

### Selection of Priors

For the Beta-coalescent we parametrize the measure characterizing the genealogical prior as:


$$\begin{array}{ccc} \text{Λ}=\nu Beta(1-\alpha^{*},1+\alpha^{*}) & & \end{array}$$ (5)

ν and $\alpha^{*}$ are unknown parameters we wish to infer. $\nu \in R_{+}$ is the time scale of the process and $\alpha^{*}\in [0,1]$ controls the Beta distribution governing the merger sizes. $\alpha^{*}$ relates to the original parameter α from Equation 3 (Schweinsberg  2003) as $\alpha =\alpha^{*}+1$. We equip these parameters with the following prior distributions:


log ⁡ ν∼Normal(0,4)α∗∼Beta(3,1) (6)

We caution that it is not straightforward to interpret ν as the timescale of the Beta-coalescent is $1∕N^{1-\alpha }$ and therefore ν only corresponds to the usual effective population size if α=2. The prior above on ν represents a weakly non-informative choice for the coalescent timescale. The prior above on $\alpha^{*}$ puts approximately 50% of prior mass on values of $\alpha^{*}>0.8$, therefore prioritizing trees that mostly contain binary mergers. For the extended Beta-coalescent, we parameterize the genealogical prior as:


$$\begin{array}{ccc} \text{Λ}=\nu ((1-\phi )\delta_{0}+\phi Beta(1-\alpha^{*},1+\alpha^{*})) & & \end{array}$$ (7)

$\nu \in R_{+}$ corresponds to the process rate, ϕ∈[0,1] is the mixing proportion between the Kingman component and the Beta component, and $\alpha^{*}\in [0,1]$ once again controls the Beta distribution and therefore the size of multiple mergers. We equip these parameters with the following prior distribution:


log ⁡ ν∼Normal(0,4)ϕ∼Beta(1,3)α∗∼Beta(1,2) (8)

Note that compared to Equation 6 we used a prior on $\alpha^{*}$ which gives less weight to the Kingman limit at $\alpha^{*}=1$ since this is also obtained in the mixture when ϕ is close to zero. This prior above is also only weakly informative if there is strong evidence of multiple mergers. As we expected Kingman-like behavior to be more common than multiple mergers, we chose the prior above on ϕ as it puts approximately 50% of mass on values of ϕ>0.8. The priors on $\alpha^{*}$ and ϕ can have a significant impact on results, which can be monitored by comparing the posterior and prior distributions of these parameters, and by performing prior sensitivity analyses as required.

Finally, for the molecular clock model, we use the ARC (Didelot et al.  2021) in which a branch of length l carries a number of substitutions x distributed as:


$$\begin{array}{ccc} x∼NegBin\left(\frac{\mu l}{\omega },\frac{\omega }{1+\omega }\right) & & \end{array}$$ (9)

where μ represents the mean clock rate and ω controls the amount of relaxation relative to a strict clock model. We use the following prior distribution:


$$\begin{array}{ccc} \begin{array}{ccc} \mu & ∼Gamma(2,8) & \\ \omega & ∼Normal(0,2)1_{\left[0,\infty \right)} \end{array} & & \end{array}$$ (10)

This prior on the mean clock rate has 95% of its weight on values between 2 and 44 substitutions per genome per year, while the prior on the relaxation parameter is concentrated on 0 while allowing values up to 5. This choice of priors covers values of the same order as was previously reported when using the ARC on several real data sets (Didelot et al.  2021).

### Billera-Holmes-Vogtmann Space Embedding

In order to efficiently sample genealogies that admit multiple mergers, we leverage 2 concepts. First, we embed binary trees in a space of phylogenetic trees where coordinates correspond to branch lengths, specifically the BHV)space. Second, we use a spike-and-slab construction to put positive mass on the set of trees where at least 1 branch length is shrunk to exactly 0 and identify these trees with multiple merger trees obtained by collapsing all branches with lengths equal to exactly 0. The BHV space is useful for 2 main reasons. First, it allows us to identify trees with multiple mergers as a subset of space of fully resolved trees, and therefore, justify a spike-and-slab construction. Second, its structure assists with the calculation of weighting terms associated with the embedding we use.

BHV space is a metric space introduced in the study of phylogenetic tree geometry (Billera et al.  2001). For a fixed number of tips n the BHV space is constructed from a set of (2n−3)!! orthants in ${\left(0,\infty \right)}^{n-2}$ each corresponding to a particular topology of a rooted binary tip labeled n-tree. Each coordinate within the n−2 dimensional orthant corresponds to the length of an interior branch in the given tree topology. At any of the 0 boundaries, the binary topology degenerates into a tree that has branching greater than 2. The orthants are “glued” together at boundaries corresponding to the same topology. For example if only 1 coordinate approaches 0 there is a junction of 3 different n−2 dimensional orthants corresponding to an n−3 dimensional orthant face. For existence of centroids, proof of constant negative curvature and properties of geodesics refer to (Billera et al.  2001). We will base our sampling scheme construction around the BHV space. The BHV space has been used previously in phylogenetic reconstruction for example to construct an embedding amenable to sampling binary trees using discrete Hamiltonian Monte Carlo (Dinh et al.  2017). Typically such approaches simply select the base measure to be Lebesgue within each of the n−2 dimensional orthants corresponding to binary topologies, and each n−2 dimensional orthant is assigned equal probability. The lower dimensional orthants are then null sets within this measure space. However, such an approach is not appropriate for inference with multiple merger trees as any set consisting purely of these would be given a 0 probability. One option would be to put mass on lower dimensional orthants, and set up a trans-dimensional sampling scheme using reversible jump MCMC (Green  1995). However this would be unlikely to work efficiently as designing moves that remove or add more than 1 branch at a time and do not suffer from a rapidly diminishing acceptance ratio would be challenging, and would not take advantage of the natural geometry of the space.

We will therefore augment the BHV space using a spike-and-slab construction (George and McCulloch  1993). Denote by T the set of all rooted, labeled, metric n-trees. An n-tree is said to be metric if all of its branch lengths are strictly greater than 0. Let 𝕋 denote the set of all labeled, rooted, binary n-tree topologies. Denote the closed n−2 dimensional orthant of the BHV space corresponding to a particular binary n-tree topology as $V^{\tau }$ for τ∈𝕋. We identify points in the closed n−2 dimensional orthants of BHV space by the tuple (X,Q,τ) where $X\in [0,\infty {)}^{n-2}$ denotes the location within an orthant, $Q\in {\left\{0,1\right\}}^{n-2}$ is a vector of indicators where $q_{i}=1$ if and only if $x_{i}=0$ and τ∈𝕋 denotes the orthant index, that is, the corresponding binary topology. We next identify all points on the boundary $\partial V^{\tau }$, that is all points with at least 1 coordinate equal to zero with the corresponding k-ary metric n-tree by collapsing each and every branch for which $q_{i}=1$, that is of length exactly 0. We now define the base measure to be


$$\begin{array}{ccc} \nu (dx\times d\tau )=\delta_{\tau }(d\tau )⊗\mu (dx) & & \end{array}$$ (11)

That is we assign uniform mass to each of the n−2 dimensional orthants and within each orthant we have


$$\begin{array}{ccc} \mu ={\left(\delta_{0}+\mu_{0}\right)}^{n-2} & & \end{array}$$ (12)

Where $\delta_{0}$ is an atom at zero and $\mu_{0}$ is the Lebesgue measure on [0,∞). This construction assigns positive probability to sets of binary trees with 1 or more branches with length exactly 0. By identifying such trees with a (metric) multiple merger tree we can see that it therefore puts a positive probability on trees with multiple mergers.

### Parametrization of the Genealogy

Having outlined the construction above we now give an explicit parametrization of the genealogy that will allow us to construct an MCMC scheme as well as enable the computation of necessary quantities. As an input we assume we are given a rooted binary phylogeny with n tips, with branch lengths corresponding to the estimated number of substitutions along a branch. This phylogeny is assumed to be a point estimate obtained by ML phylogenetic software. The root position may be assumed to be known a priori, or to be estimated, in which case the initial rooting is assumed to be chosen arbitrarily. In practice, the estimated number of substitutions along a branch may not be an integer even though it is likely to be very close to an integer when the number of substitutions per site is low. The clock models used require an integer number of mutations. Therefore, all branch lengths are coerced to integer values by rounding. Based on the undated input phylogeny, we can construct a rooted binary genealogy denoted as τ as follows. We define the following notations:


$$\begin{array}{cccc} \{1,2,...,2n-1\}\; & & \text{Set of all nodes}\\ S:=\{1,2,...,n\}\; & & \text{Set of tips} & \\ I:=\{n+1,n+2,...,2n-1\}\; & & \text{Set of internal nodes} & \\ \{2n-1\}\; & & \text{Root node} & \\ {pa}_{\tau }(i),i\in \{1,...,2n-2\}\; & & \text{Parent of node }i & \\ m_{\tau }(i),i\in \{1,...,2n-2\}\; & & \text{Number of mutations on edge above node }i & \\ s_{\tau }(i),i\in S\; & & \text{Sampling date of tip }i & \\ j{≺}_{\tau }i\; & & \text{Node }j\text{ is a descendant of node }i & \\ {desc}_{\tau }(i):=\left\{j\in S:j{≺}_{\tau }i\right\}\; & & \text{Set of leaves descendant of node }i & \\ C_{\tau }:=\{c_{l},c_{r}\}\; & & \text{Children of the root node} \end{array}$$

Having defined necessary notation for characterizing the rooted tree topology we aim to define and parametrize the internal node heights in the rooted genealogy. This corresponds to the within orthant parametrization. We now proceed to define the collection of node times $t:={\left\{t_{i}\right\}}_{1\leq i\leq 2n-1}$. A subset of these variables corresponds to the internal node times, that is the free parameters of the model. We denote these as $t^{I}:=\{t_{i}\in t:i\in I\}$. The remaining first n variables correspond to sampling times and these are considered as an input. Denote this set of sampling times by $t^{S}:=\{t_{i}\in t:i\in S\}$. In practice, the vector t needs to satisfy a complex set of constraints, that is


$$\begin{array}{ccc} i{≺}_{\tau }j\;⟹\;\{\begin{array}{cc} t_{i}\leq t_{j}\; & \;i>n\\ t_{i}<t_{j}\; & \;i\leq n\\ \; \end{array} & & \end{array}$$ (13)

Where $i{≺}_{\tau }j$ denotes the partial order induced by the tree topology, that is $i{≺}_{\tau }j$ if and only if i is a descendant of j. Note that if i is an internal node then $t_{i}=t_{j}$ is possible if the nodes i and j belong to the same multi-merger event.

The complex set of constraints that the node times have to satisfy would make defining a Metropolis-Hastings move capable of targeting the boundaries complicated. Hence, we define the vector of positive height variables $\chi \in {\mathbb{R}}_{+}^{n-2}$ as well as the tree height variable H∈ℝ (which can be negative as it is log-transformed). Once again for convenience we will abuse notation and denote by χ(i) the component of χ associated with node i. This is in contrast to $\chi_{i}$ which denotes the ith component of the vector χ. To define the transformation for χ to t, we first need to introduce the set of lower bounds $b_{\tau }$


bτ(i)= max ⁡ j:descτ(i)sτ(j),i∈[2n−1] (14)

We now map χ,H to t via the mapping g:(H,χ)↦t. The first part of the mapping parametrizes the height of the root node by transforming H


$$\begin{array}{ccc} t_{2n-1}=e^{H}+b_{\tau }(2n-1) & & \end{array}$$ (15)

The second part of mapping parametrizes the height of internal non-root nodes by transforming χ


$$\begin{array}{ccc} \begin{array}{c} t_{i}=(t_{{pa}_{\tau }(i)}-b_{\tau }(i))e^{-\chi (i)}+b_{\tau }(i) \end{array} & & \end{array}$$ (16)

**Figure 1.**
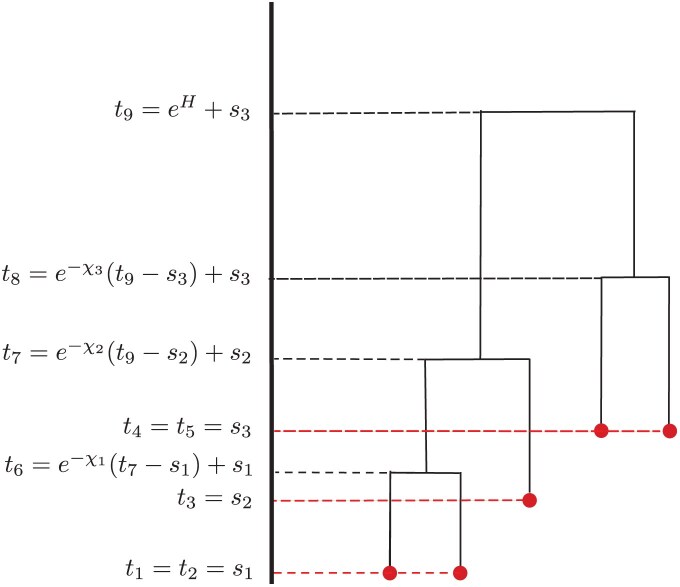
Example of tree parametrization. In this case, the bounds are $b(6)=s_{1},b(7)=s_{2},b(8)=s_{3},b(9)=s_{3}$. The components χ are mapped as $\chi (6)=\chi_{1},\chi (7)=\chi_{2},\chi (8)=\chi_{3}$.

Note that χ(i)=0 implies that the length of the branch above node i scaled in time units is also 0. An illustration of how such parametrization looks in practice can be found in Figure 1.

This parametrization is reminiscent of the ratio transform of (Ji et al.  2023), with the key difference that we express non-root internal node heights in terms of a transform of the distance from the parent constrained so that the age of the child is strictly less than the oldest descendant tip as opposed to a transform of the ratio of the remaining height. This is because it is necessary in the construction presented here for the boundary corresponding to 0 branch length to be accessible. Finally we introduce the vector of indicators that determine whether a coordinate is allocated to the boundary:


$$\begin{array}{ccc} \begin{array}{ccc} q & \in {\left\{0,1\right\}}^{n-1} & \\ q(i)=1 & \Leftrightarrow \chi (i)=0 \end{array} & & \end{array}$$ (17)

We can now parametrize a genealogy by the tuple Y:=(H,χ,q,τ;s), where τ specifies the binary topology and hence a full-dimensional orthant of the BHV space, and H,χ,q characterize the position within the orthant conditional on the tip date constraints s.

### Lambda-Coalescent Likelihood

In order to compute the coalescent likelihood of a genealogy, we first need to convert the (possibly non-metric) binary tree embedding to the respective metric multiple merger tree. To achieve this first denote the set of all internal nodes descending from node i as


$$\begin{array}{ccc} r(i):=\{j\in [2n-1]:j{≺}_{\tau }i\} & & \end{array}$$ (18)

Next defining the set of internal descendants of i coincident with i as


$$\begin{array}{ccc} z(i):=\{j\in r(i):t(i)=t(j)\} & & \end{array}$$ (19)

We can see that for any internal node i such that q(i)=0, |z(i)| corresponds to the merger size minus 2. Using these 2 definitions, we can compute the likelihood of the binary tree embedding Y:=(H,χ,q,τ;s) under the Lambda-coalescent ${\text{Λ}}^{\theta }$ with block merger rates $\lambda_{b,k}^{\theta }$ as:


$$\begin{array}{ccc} \begin{array}{ccc} f_{\theta }^{\text{Λ}}(Y) & =\underset{\begin{array}{c} i\in I:\\ q_{i}=0 \end{array}}{\prod }\lambda_{A(t_{i}),|z(i)|+2}^{\theta } & \\ & \times \underset{\begin{array}{c} 1<i\leq 2n-1:\\ q_{i}=0 \end{array}}{\prod }e^{-R(A(t_{i}))(t_{i}-\max\{t_{j}:t_{j}<t_{i}\})}\\ & \times J_{g}{|}_{Y}\\ & \times \frac{1}{Z(Y)} \end{array} & & \end{array}$$ (20)

Where $R:N\mapsto R_{+}$ denotes the total coalescent rate of the process


R(b):= ∑k=1bbkλb,kθ (21)

and $A:R_{+}\mapsto Z_{+}$ denotes the lineages through time function at time t, defined as


$$\begin{array}{ccc} A(t):=\underset{i\in S}{\sum }1_{t>t_{i}}(t_{i})-\underset{i\in I}{\sum }1_{0}(q_{i})1_{t>t_{i}}(t_{i})(|z(i)|+1) & & \end{array}$$ (22)

This is also known as the block counting process (Kukla and Möhle  2018). As the genealogical prior is expressed in terms of node heights t, we must account for the transformation from χ to t in the density. To do so, we require the Jacobian for the mapping g:(H,χ)↦t. This is straightforward to compute as the matrix of first order partials has a diagonal structure and hence is equal to


$$\begin{array}{ccc} J_{g}{|}_{Y}=e^{H}\underset{i\in I}{\prod }\left[1_{0}(q(i))(t_{{pa}_{\tau }(i)}-s_{\tau }(i))e^{-\chi (i)}+1_{1}(q(i)))\right] & & \end{array}$$ (23)

Note that as the density of all $\chi_{i}=0$ is with respect to an atomic measure these do not play a role within the relevant Jacobian adjustment. Finally, we note that the embedding of a given multimerger tree as a binary tree is not unique. Therefore, in order for the density of a given tree to be proportional to the density given by the Lambda-coalescent density, we need to re-weight the density of the embedding to account for the overcounting. In general, given a multiple merger of size m, there are (2m−3)!! ways to resolve this as a sequence of binary mergers. This is the number of rooted labeled binary trees with m tips (Billera et al.  2001). Therefore, the adjustment for the embedding Y is:


$$\begin{array}{ccc} Z(Y)=\underset{i\in I}{\prod }(2|z(i)|+4-3)!! & & \end{array}$$ (24)

### Branch Likelihood

The likelihood is based on the ARC model (Didelot et al.  2021). Each branch in the input ML phylogeny is scaled in estimated number of substitutions along that branch. This number of substitutions is assumed to be distributed according to Equation 9. ML software usually estimate branch lengths as a continuous value, and since the ARC model requires a discrete number of substitutions, all values are rounded to the nearest integer. Although this rounding introduces a slight approximation, in practice the branch lengths are often close to an integer value, especially when they are short (Didelot et al.  2021). Define the length of a branch above node i as $l(i)=t_{{pa}_{\tau }(i)}-t_{i}$. Given the current genealogy state parameterized by Y, and the clock parameters μ and ω, the likelihood for all branches not incident to the root can be computed as:


$$\begin{array}{ccc} \underset{\begin{array}{c} i\in I:\\ i\notin C_{\tau }\cup \{2n-1\} \end{array}}{\prod }NegBin\left(m_{\tau }(i);\frac{\mu l(i)}{\omega },\frac{\omega }{1+\omega }\right) & & \end{array}$$ (25)

This has to be multiplied by the likelihood of the branches incident to the root. This depends on whether the root is considered fixed, or if it is unknown. If the root is fixed, the branches incident to root are treated as any other branch and their contribution is


$$\begin{array}{ccc} \underset{i\in C_{\tau }}{\prod }NegBin\left(m_{\tau }(i);\frac{\mu l(i)}{\omega },\frac{\omega }{1+\omega }\right) & & \end{array}$$ (26)

If the root position is considered to be unknown then the position of the root on the branch is marginalized out and the contribution becomes


$$\begin{array}{ccc} NegBin\left(m_{\tau }(c_{l})+m_{\tau }(c_{r});\frac{\mu (l(c_{l})+l(c_{r}))}{\omega },\frac{\omega }{1+\omega }\right) & & \end{array}$$ (27)

This follows from the additive nature of the ARC clock model (Didelot et al.  2021).

### Implementation

The MCMC scheme used to perform Bayesian inference is described in the Supplementary material. We implemented the approach described in a new R package entitled *MMCTime*, which is available at https://github.com/dhelekal/MMCTime. The package uses *ape* (Paradis and Schliep  2019) as a backend for handling phylogenies. *Bayesplot* is used for handling MCMC diagnostic visualizations, (Gabry et al.  2019) and *ggtree* (Yu et al.  2017) is used for visualizing phylogenies. The package *posterior* (Vehtari et al.  2021) is used for computing MCMC diagnostics.

## RESULTS

### Illustration on Simulated Phylogenies

All simulations follow the same protocol. First, a dated phylogeny is simulated from a genealogical model, conditional on appropriate parameter values and tip sample times. Then, the expected number of substitutions for each branch is sampled using the ARC model (Didelot et al.  2021). Then, Seq-Gen (Rambaut and Grassly  1997) is used to generate sequences of a given length under the HKY model (Hasegawa et al.  1985). For all simulation experiments, the length was set to 10,000 bp, except if otherwise mentioned. Finally, an undated ML phylogenetic tree is reconstructed using IQ-TREE (Minh et al.  2020). This, along with the tip dates, serves as the input for the dating analysis. For all simulation benchmarks, the root position was assumed to be unknown and to be inferred.

**Figure 2.**
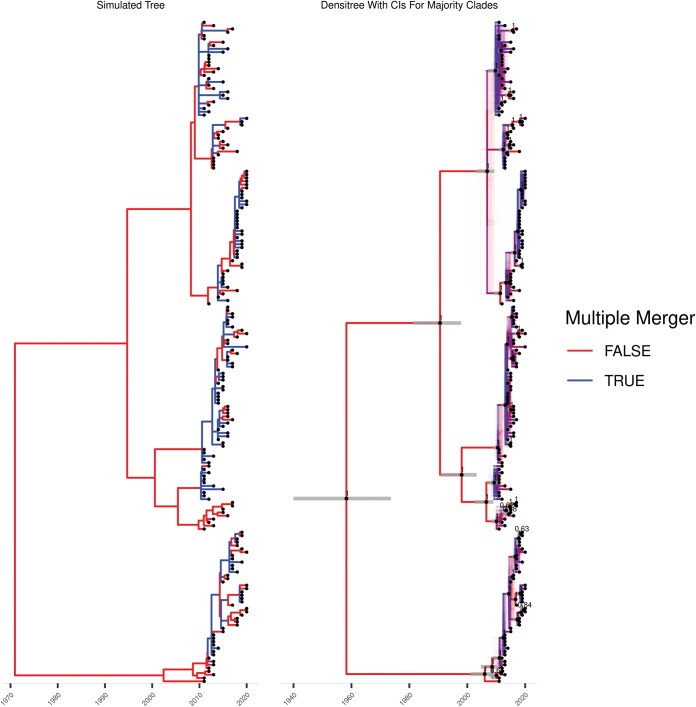
Simulation and inference under the Beta-coalescent model. (A) The simulated phylogeny. (B) A qualitative summary of the posterior, showing locations of possible multiple mergers and uncertainty in polytomy topology. Clades appearing in over 50% of posterior samples are indicated with black dots fixed at median height, and gray bars overlayed indicated the 95% posterior credible interval for the height of these nodes.

A first dated phylogeny was simulated under the Beta-coalescent (Equation 5) with parameters $\left\{\nu =1\text{∕}12,\;\alpha^{*}=1\text{∕}2\right\}$ as shown in Figure 2A. The ARC clock model with parameters {μ=1,ω=1} was applied, and the input ML phylogeny is shown in Supplementary Figure S1.Inference was performed under the Beta-coalescent model using 4 chains, sampling every 2000 iterations for a total of 1000 samples retained per chain. Assessing mixing and qualities of estimates is challenging in this setting as the topology changes due to the uncertain branching on polytomies. Furthermore, standard metrics like the Robinson-Foulds distance (Robinson and Foulds  1981) were not designed for dated trees and are not applicable to multiple merger trees. To circumvent this, we compute effective sample sizes (ESS) and $\hat{r}$ estimates for the genealogical parameters as well as the clock parameters along with the tree height and the 2 following summaries: the number of multiple mergers and the maximum merger size. The $\hat{r}$ and ESS estimates are computed using technique in (Vehtari et al.  2021) as implemented in the R package posterior. This confirmed that the MCMC had converged and mixed as expected. The MCMC traces are shown in Supplementary Figure S2 and the inferred parameters in Supplementary Figure S3, with all inferred ranges covering the correct values. Nine posterior samples of the dated phylogeny are shown Supplementary Figure S4. To summarize the full posterior sample of dated phylogenies, we use a modified version of the DensiTree representation (Bouckaert  2010) as shown in Figure 2B. Comparison of the simulated (Fig. 2A) and inferred (Fig. 2B) phylogenies demonstrate the accuracy of the inference, including the identification of which nodes are likely to be multiple merger events. The average branch score distance (Kuhner and Felsenstein  1994) between the simulated and inferred trees was 26.35.

**Figure 3.**
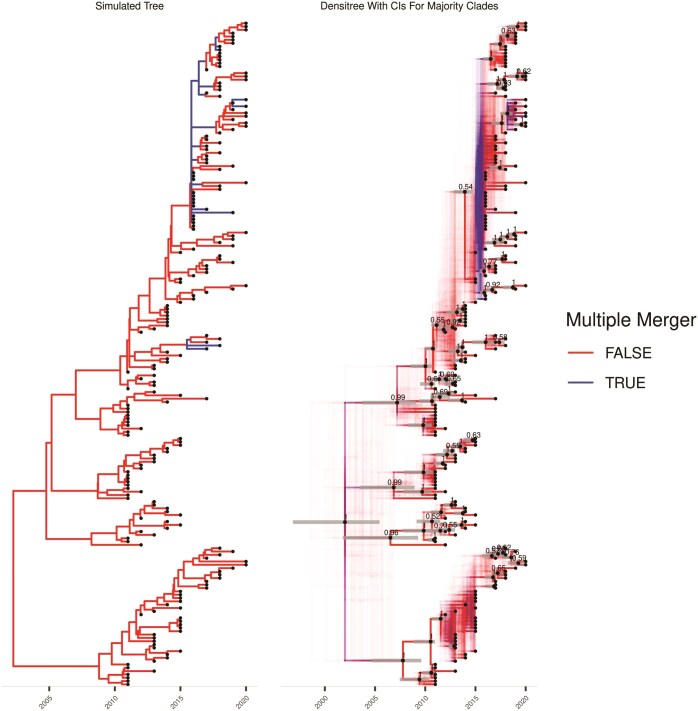
Simulation and inference under the extended Beta-coalescent. (A) The simulated phylogeny. (B) A qualitative summary of the posterior, showing locations possible multiple mergers and uncertainty in polytomy topology. Clades appearing in over 50% of posterior samples are indicated with black dots fixed at median height, and gray bars overlayed indicated the 95% posterior credible interval for the height of these nodes.

Next a dated phylogeny was simulated under the extended Beta-coalescent (Equation 7) with parameters $\left\{\nu =1\text{∕}12,\;\alpha^{*}=1\text{∕}5,\;\phi =2\text{∕}5\right\}$ (Fig. 3A). The same analysis as above was performed, except that the extended Beta-coalescent model was used for inference. The ML tree is shown in Supplementary Figure S5, the MCMC traces in Supplementary Figure S6, the parameters in Supplementary Figure S7, 9 posterior sampled phylogenies in Supplementary Figure S8 and the posterior phylogeny summary in Figure 3B. Once again we find that the parameters and phylogeny are inferred satisfactorily. There were only a few multiple merger events in the simulated dated tree, most of which behaved as a Kingman’s coalescent tree. This represents a good illustration of what can be achieved with the extended Beta-coalescent, and would be very unlikely to happen under the Beta-coalescent model. The average branch score distance (Kuhner and Felsenstein  1994) between the simulated and inferred trees was 16.1. In particular, the root of the tree was binary in the simulation (Fig. 3A) but was given a probability of 39% to be a multimerger (Fig. 3B).

**Figure 4.**
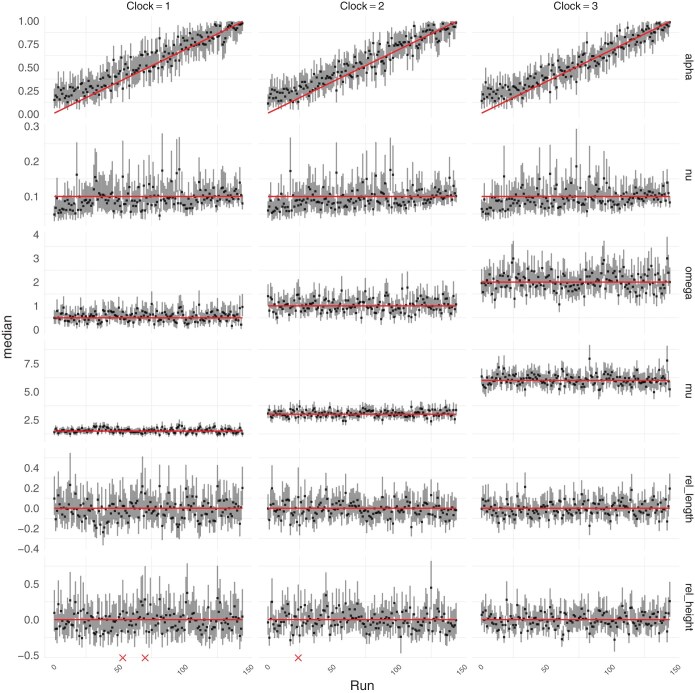
Simulation benchmark under the Beta-coalescent. Posterior summaries for analysis of limitations of the likelihood approximation. Red lines indicate ground truth. Vertical bars represent 95% posterior credible intervals, and points represent the median. Red crosses indicate insufficient mixing in the corresponding.

### Benchmark Under the Beta-Coalescent

To benchmark the performance of inference under the Beta-coalescent, we considered 3 ARC clocks with the following parameters: Clock 1 has μ=1.5 and ω=0.5, Clock 2 has μ=3 and ω=1.0, and Clock 3 has μ=6 and ω=2. Supplementary Figure S9 shows the distributions of the number of substitutions per site under each of these clock models. For each clock model, Figure 4 shows the results of parameter inference under the Beta-coalescent for 150 data sets generated with ν=0.1 and values of $\alpha^{*}$ increasing between 0 and 1. In every case the parameters are correctly inferred, except for $\alpha^{*}$ which is slightly overestimated when the correct value was lower than 0.1. This is likely to be caused by our use of a Beta(3,1) prior in $\alpha^{*}$, cf Equation 6, which only has cumulative probability 0.001 for $\alpha^{*}\in [0,0.1]$. Low values of $\alpha^{*}$ lead to trees with a high probability of large multiple merger events which are biologically implausible. Furthermore this prior gives more weight to high values of $\alpha^{*}$ close to 1, which corresponds to the limit where the Beta-coalescent reduces to the Kingman coalescent. In other words, this choice of prior makes our inference method relatively conservative in terms of the detection of multiple merger events, which needs to be guided by evidence in the data.

Supplementary Figure S10 shows the number of nodes in the inferred phylogenies minus the true number of nodes in the simulated phylogenies. Positive values indicate an excess of nodes and therefore an underestimation of the number of multiple merger events. Negative values indicate a lack of nodes and therefore an underestimation of the number of multiple merger events. Most intervals cover the correct value of 0. There is a slight tendency to overestimate the number of nodes overall, which is again driven by our use of a conservative prior for $\alpha^{*}$.

**Figure 5.**
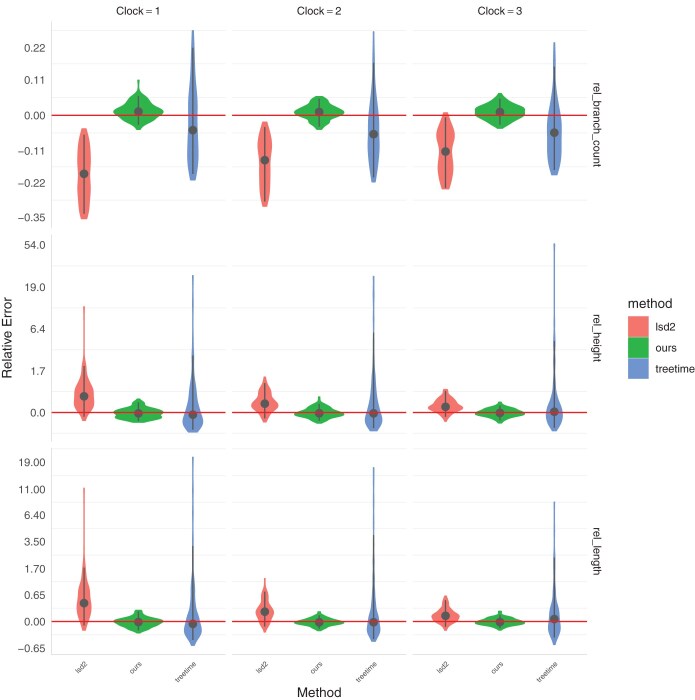
Comparison of branch count (top), tree height (middle), and tree length (bottom) estimated by LSD2, TreeTime, and our method for the simulation benchmark under the Beta-coalescent.

We use 3 metrics to assess the quality of reconstructed dated tree: the relative branch count, relative tree height and relative tree length. The relative branch count is the number of inferred branches of non-zero length divided by its correct value. The relative tree height is the age of the root in the inferred tree divided by its correct value. The relative tree length is the sum of all branch lengths in the inferred tree, divided by its correct value. Figure 5 and Table 1 show a comparison between our method, LSD2 (To et al.  2016) as implemented in the R package rLsd2 and TreeTime (Sagulenko et al.  2018). LSD2 was run with the option estimateRoot=’a’ option. TreeTime was run with options –coalescent const –max-iter 150 –relax 5.0 0 –time-marginal false. As both LSD2 and TreeTime are point estimators, we need to summarize the output of our method as point estimate as well. We use the median of the posterior for this purpose. Both LSD2 and TreeTime overestimated the number of branches of length 0, which presumably stems from the fact that any branch with no substitutions would have highest likelihood to occur if the duration of the length was 0. This comparison is not entirely fair since our method is the only one to be aware of the possibility of multiple mergers events. The comparison on tree height and tree length on the other hand is more informative since it demonstrates that ignoring multiple merger events can result in significant bias on both of these quantities. Furthermore, we focused on the point estimates computed by each method whereas the confidence intervals are sometimes wide, providing clues that the point estimates may not be reliable.

### Benchmark Under the Extended Beta-Coalescent

We performed a similar benchmark for the inference under the extended Beta-coalescent. Four scenarios were considered with ϕ∈{0,0.5,0.75,1}. Each scenario consists of 48 genealogies. Within a scenario the values of $\alpha^{*}$ were linearly varied from $\alpha^{*}=0.01$ to $\alpha^{*}=0.75$. The upper limit was chosen to 0.75 since as $\alpha^{*}\to 1$ the process behaves like Kingman’s coalescent irrespective of the value of ϕ, rendering the scenario meaningless. As for the previous benchmark the genealogies were then used to generate 3 data sets each with different clock parameters. Supplementary Figure S11 shows the distributions of the number of substitutions per site under each of the 3 clock models.

The inferred values of the parameters ϕ and $\alpha^{*}$ are shown in Supplementary Supplementary Figure S12 and S13, respectively. The scenario ϕ=0 corresponds to the Kingman’s coalescent. In this scenario, the parameter ϕ was consistently estimated to be low as expected, and $\alpha^{*}$ could not be estimated (i.e., the posterior was approximately equal to the prior) since in this scenario this parameter does not play a role. In the scenarios where the Kingman’s coalescent and Beta-coalescent were mixed with ϕ=0.5 and ϕ=0.75 the parameter ϕ was usually underestimated to the point that the inferred values of $\alpha^{*}$ did not follow the correct values. However, in the scenario ϕ=1, which corresponds to the pure Beta-coalescent, it was possible to infer the values of ϕ and $\alpha^{*}$ as long as $\alpha^{*}$ was not too high. When $\alpha^{*}$ is high the Beta-coalescent component of the extended Beta-coalescent prior behaves like the Kingman’s coalescent component, so that the mixing proportion ϕ does not have much effect on the data.

Thus the extended Beta-coalescent suffers from identifiability issues on the parameters ϕ and $\alpha^{*}$ in the part of the parameter space where the model reduces to the Kingman’s coalescent, namely when ϕ is low and/or $\alpha^{*}$ is high. This does not affect the estimates of the remaining parameters though. The parameters ν, μ and ω are shown in Supplementary Figures S14, S15, and S16, respectively, and are all well estimated. Supplementary Figures S17, S18, and S19, show that the tree height, tree length and number of branches in the tree are also estimated around their correct values. Note that in the scenario with ϕ=0 the correct tree is completely binary and so the number of nodes can only be underestimated. Supplementary Figure S20 shows the estimated probabilities that a tree sampled from the posterior contains a multiple merger, which increases as expected as ϕ increases.

Comparisons with LSD2 and TreeTime on the estimation of the tree height, tree length and number of branches under the extended Beta-coalescent are shown in Supplementary Figures S21, S22, and S23, respectively. These comparisons for the Beta-coalescent model are summarized in Supplementary Table S1. It is also interesting to compute the distance between the correct simulated dates tree and the ones inferred by the different methods. We use the branch score distance as metric since it has the advantage to account for branch lengths and apply to both binary and non-binary nodes (Kuhner and Felsenstein  1994). The results comparing LSD2, TreeTime, and our methods are shown in Supplementary Figure S24 for the Beta-coalescent simulations and in Supplementary Figure S25 for the extended Beta-coalescent simulations. These results are also summarized in Supplementary Table S2. An example of Beta-coalescent simulation where both LSD2 and TreeTime had high errors compared to our method is shown in Supplementary Figure S26. We note that the rooting of the tree was correctly performed by all methods. The errors likely resulted from clock overdispersion exacerbated by the presence of polytomies.

**Table 1.** Comparison of branch count, tree height, and tree length estimated by LSD2, TreeTime, and our method for the simulation benchmark under the Beta-coalescent.

	Avg. bias	RMSE	90% Quantile range	Statistics
LSD2	–0.153	0.175	0.267	Relative branch count
	0.385	0.769	1.12	Relative tree height
	0.323	0.710	1.01	Relative tree length
TreeTime	–0.0432	0.107	0.330	Relative branch count
	0.578	3.52	2.82	Relative tree height
	0.344	1.80	1.84	Relative tree length
Ours	0.0105	0.0249	0.0752	Relative branch count
	–0.0118	0.120	0.406	Relative tree height
	–0.00973	0.0783	0.253	Relative tree length

### High Mutation Rate Limitation

**Figure 6.**
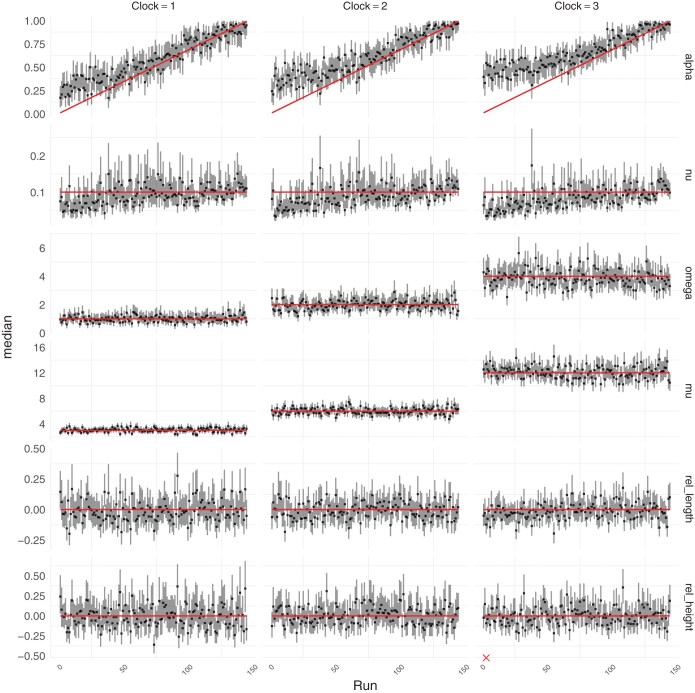
Posterior summaries for analysis of limitations of the likelihood approximation. Red lines indicate ground truth. Vertical bars represent 95% posterior credible intervals, and points represent the median. Red crosses indicate insufficient mixing in the corresponding run.

A difficulty arises when the mutation rate per site is too high. In this case, the probability of reversal or homoplasious mutation increases, such that the maximum likelihood estimated branch lengths of the input phylogeny become unlikely to be exactly 0 even when a multiple merger event occurred. This is related to the branch saturation observed in dating methods that use a maximum likelihood tree as input, such as LSD (To et al.  2016) or BactDating (Didelot et al.  2018). In order to gain an understanding of when this phenomenon becomes problematic, we repeated the simulation benchmark under the Beta-coalescent but with a genome length of 1000 bp (i.e., 10 times less than previously) and mutation rates doubled for each clock model, that is μ∈{3,6,12}. Figure 6 shows the results for this analysis. There is a clear and consistent overestimation of $\alpha^{*}$ when the correct value of this parameter was low. This corresponds to bias against configurations of the Beta-coalescent that produce larger multiple mergers. This was accompanied by underestimation of the process timescale parameter ν. This bias worsens as the mutation rate increases and thus the expected number of substitutions per site increases. This result shows that the approach presented here is not appropriate when the number of substitutions per site is too high. Specifically, when μ≈10 per year on trees with sums of branch lengths in excess of 100 years (e.g., Fig. 2A) and sequences of length 1000 bp, this corresponds to an expected number of substitutions around 1 for each site. In this situation, standard phylogenetic methods typically underestimate the branch lengths, but this is not an issue since in practice we consider situations where there is much less than 1 substitution per site.

**Figure 7.**
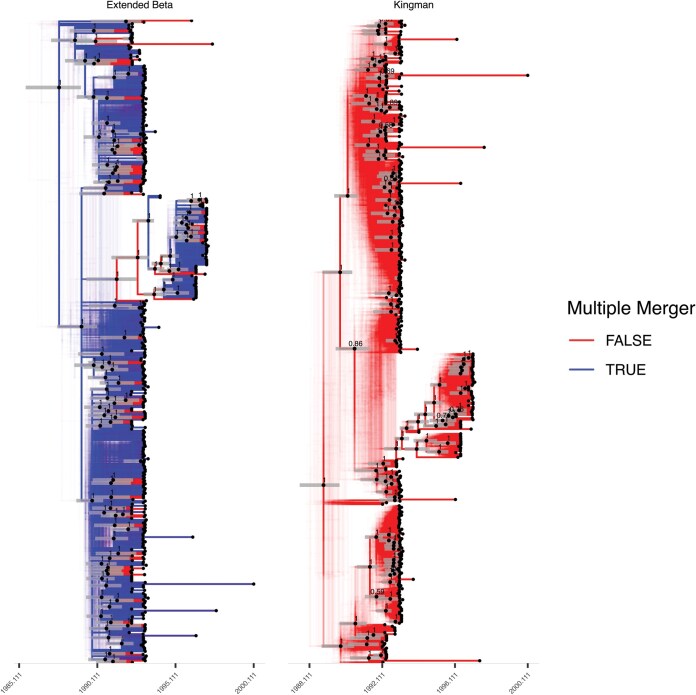
Analysis of the *Vibrio cholerae* data set. Qualitative summaries of the posterior inferred under the extended Beta-coalescent and Kingman’s coalescent, showing locations possible multiple mergers and uncertainty in polytomy topology. Note that the tip ordering is not identical between the 2 summaries. Clades appearing in over 50% of posterior samples are indicated with black dots fixed at median height, and gray bars overlayed indicated the 95% posterior credible interval for the height of these nodes.

### Case Study: Spread of Vibrio cholerae in Argentina

A recent study compared genome sequences of *Vibrio cholerae*, the causative agent of cholera, sampled from Argentina and neighboring countries between 1992 and 2000 in order to characterize its population structure (Dorman et al.  2020). We selected from the previously published phylogeny the genomes that had been isolated in Argentina and for which the isolation date was known, resulting in a phylogeny containing 411 leaves as shown in Supplementary Figure S27. We applied inference under the extended Beta-coalescent model, which produced the traces shown in Supplementary Figure S28 and the parameter estimates shown in Supplementary Figure S29. The rooting of the tree was fixed using an outgroup. Nine samples from the posterior phylogeny are shown in Supplementary Figure S30. A phylogenetic posterior sample is summarized as a DensiTree in Figure 7. This contains several large well supported multiple merger events, consistent with the high estimate of ϕ and low estimate of $\alpha^{*}$ (Table 2).

For comparison purposes, we also performed inference under the pure Kingman’s coalescent model, and 9 samples from the posterior phylogeny are shown in Supplementary Figure S31. As we can see in Table 2 the clock rate estimated for the *Vibrio cholerae* genealogy under Kingman’s coalescent is much higher than the 1 under the extended Beta-coalescent. Furthermore, the relaxation parameter ω is higher when using the Kingman’s coalescent, indicating that evolution is less clock-like. The estimated mutation rate under the extended Beta-coalescent falls into a posterior 95% credible interval of [1.90–2.84] mutations per genome per year. This is in good agreement with previous estimates of the *V. cholerae* clock rate based on sparsely sampled worldwide collections of genomes (Mutreja et al.  2011; Didelot et al.  2015). In contrast, the substitution rate estimated under Kingman’s coalescent is higher with credible interval [4.98–6.64] mutations per genome per year, which is inconsistent with previous estimates. Consequently, the time to the most recent common ancestor for the whole Argentinian data set is underestimated when using Kingman’s coalescent as opposed to the extended Beta-coalescent (Table 2).

### Case Study: Mycobacterium tuberculosis Outbreak Phylogenies

The importance of multiple merger genealogies to study tuberculosis outbreaks has been recently demonstrated (Menardo et al.  2021) using data from 11 previously published outbreaks. We selected 3 of these for reanalysis, labeled *Bainomugisa2018* (Bainomugisa et al.  2018), *Eldholm2015* (Eldholm et al.  2015) and *Lee2015* (Lee et al.  2015). These 3 data sets were selected because they had more than 90% probability of the model selected being a Beta-coalescent in the previous analysis (Menardo et al.  2021) and their phylogenies were not being excessively large. Analysis was performed for each of the 3 phylogenies under 3 models: the extended Beta-coalescent, the Beta-coalescent, and the Kingman’s coalescent. The 3 input trees are shown in Supplementary Figure S32. The rooting of the trees was fixed using outgroup rooting.

**Table 2:** A summary (median, in brackets 95% posterior credible interval) of parameter estimates under different models for the *Vibrio cholerae* data set

	Variable	Summary
Extended beta	μ	2.320 (1.820, 2.950)
	ν	0.118 (0.075, 0.171)
	ω	1.469 (1.140, 1.877)
	$\alpha^{*}$	0.026 (0.001, 0.108)
	ϕ	0.998 (0.995, 1.000)
	Tree height	12.444 (11.050, 14.582)
	Tree length	942.430 (749.245, 1191.310)
Kingman	μ	5.767 (4.844, 6.801)
	ν	0.019 (0.015, 0.024)
	ω	3.238 (2.648, 3.924)
	Tree height	11.228 (10.346, 12.537)
	Tree length	378.219 (328.262, 442.702)

**Table 3:** A summary (median, in brackets 95% posterior credible interval) of parameter estimates under different models for each of the 3 tuberculosis data sets (from left to right *Bainomugisa2018*, *Eldholm2015*, and *Lee2015*)

		Bainomugisa2018	Eldholm2015	Lee2015
Beta	μ	0.308 (0.057, 0.763)	0.185 (0.132, 0.247)	0.350 (0.276, 0.438)
	ν	0.011 (0.002, 0.035)	0.014 (0.007, 0.028)	0.019 (0.011, 0.032)
	ω	0.569 (0.148, 1.231)	0.639 (0.325, 1.055)	0.386 (0.079, 0.891)
	$\alpha^{*}$	0.365 (0.146, 0.597)	0.295 (0.140, 0.461)	0.503 (0.299, 0.710)
	Tree height	56.125 (22.247, 306.006)	84.693 (58.555, 127.247)	106.823 (83.469, 137.216)
	Tree length	1309.457 (531.212, 6940.885)	2545.669 (1949.043, 3513.807)	1353.815 (1110.545, 1671.576)
Extended beta	μ	0.307 (0.060, 0.759)	0.188 (0.134, 0.250)	0.351 (0.278, 0.442)
	ν	0.009 (0.002, 0.029)	0.016 (0.008, 0.029)	0.018 (0.011, 0.033)
	ω	0.589 (0.161, 1.229)	0.657 (0.347, 1.082)	0.397 (0.076, 0.922)
	$\alpha^{*}$	0.172 (0.010, 0.493)	0.123 (0.006, 0.329)	0.305 (0.031, 0.609)
	ϕ	0.882 (0.612, 0.974)	0.970 (0.923, 0.990)	0.816 (0.554, 0.947)
	Tree height	56.899 (22.995, 287.468)	83.134 (56.698, 124.206)	106.489 (83.355, 136.978)
	Tree length	1309.462 (537.484, 6655.092)	2511.195 (1924.334, 3413.347)	1344.334 (1095.689, 1667.751)
Kingman	μ	0.376 (0.083, 0.903)	0.256 (0.191, 0.331)	0.375 (0.294, 0.486)
	ν	0.004 (0.001, 0.010)	0.004 (0.003, 0.005)	0.011 (0.008, 0.015)
	ω	0.943 (0.382, 1.785)	1.078 (0.686, 1.610)	0.652 (0.233, 1.286)
	Tree height	57.292 (24.040, 261.148)	75.462 (53.608, 108.566)	100.950 (77.746, 130.492)
	Tree length	1069.552 (457.987, 4867.096)	1846.323 (1474.926, 2406.447)	1257.403 (1017.572, 1573.208)

**Figure 8.**
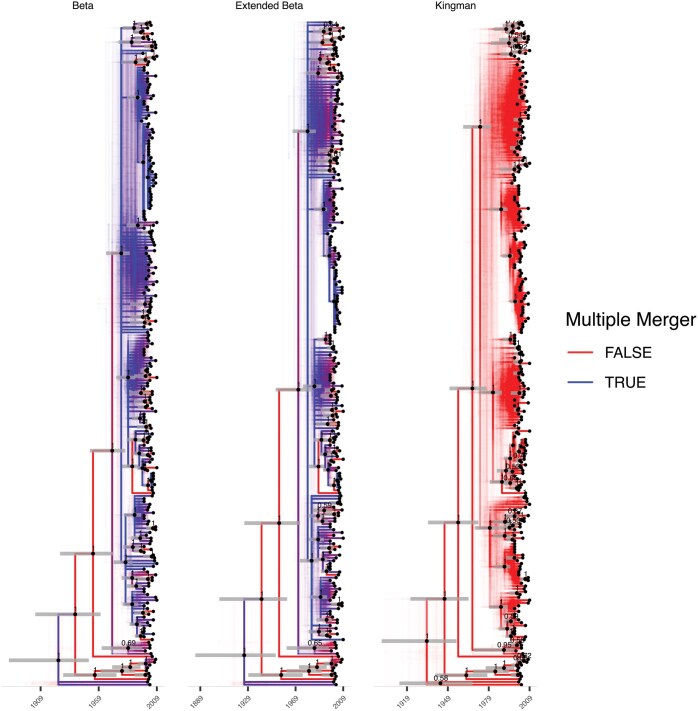
A comparison of 3 qualitative summaries of posteriors inferred for the underlying genealogies for the *Eldhom2015* data set when timed under different coalescent priors (from left to right the Beta, extended-Beta and Kingman models). Note that the tip ordering is not exactly the same between the different summaries.

As can be seen in Table 3, analysis under Kingman’s coalescent leads to a higher clock relaxation parameter ω value, suggesting that this model is less appropriate. This is also shown by the fact that $\alpha^{*}$ was always inferred much smaller than 1. The effect is most pronounced for the *Eldhom2015* data set. In the extended Beta-coalescent, the parameter ϕ was estimated to be very close to 1 for this data set, in which case it becomes approximately equivalent to the Beta-coalescent. A qualitative summary of the genealogies inferred for this data set can be seen in Figure 8. Nine realizations of the dated genealogies are shown for the Beta-coalescent, extended Beta-coalescent and Kingman’s coalescent in Supplementary Figures S33, S34, and S35, respectively. As expected, the results under the Beta-coalescent and extended Beta-coalescent are very similar, including evidence for several large multiple merger events. These results are in good agreement with the previous analysis of these 3 data sets (Menardo et al.  2021), and highlight the importance of considering multiple mergers when analyzing phylogenetic data from tuberculosis outbreaks.

## DISCUSSION

We have presented an approach to reconstructing dated phylogenies with multiple mergers under Lambda-coalescent models. To our knowledge, this is the first such approach that scales to real world phylogeny sizes, explicitly reconstructs the underlying multiple merger genealogy, and does not rely on likelihood-free approaches such as Approximate Bayesian Computation. Our focus has been on the implementation of the methodology presented, extensive benchmarks, and applications to real world examples from pathogen phylogenetics. On the other hand, we have not yet addressed how this reconstruction can be used to further study of pathogen population genetics. Some implications are relatively straightforward. For example, our method could be used as a starting point to do multifurcating skyline plot analysis (Ho and Shapiro  2011). We also envisage that it will lead to other forms of studies becoming possible, for example using genomic data to learn about superspreading, outbreaks, and impacts of selection. Our methodology should be useful for dating multifurcating phylogenies containing up to about 1000 leaves. The simulated and real examples we showed contained hundreds of leaves and each took computational time of the order of a few hours on a standard computer. This is significantly more than the few seconds needed by the methods LSD2 (To et al.  2016) and TreeTime (Sagulenko et al.  2018) used for comparison.

Other Lambda-coalescents models than the ones we used have been studied or derived. While this study was only focused on the Beta-coalescent and its mixture with a Kingman’s coalescent, it is worthwhile to mention some alternatives. In principle, there is no reason why the presented methodology would not be applicable to them. A first alternative class of Lambda-coalescent is the extinction-recolonisation Dirac coalescent of (Eldon and Wakeley 2006). In the corresponding forwards in time model all large reproductive events replace a fixed proportion of the population. Each time a potential multiple merger event occurs, a biased coin with probability of heads p∈(0,1] gets flipped for every extant lineage in the process. Lineages whose coin shows heads all merge into a common ancestor. This model is noteworthy primarily because it was amongst the first to be derived, is simple, and well studied, but it may not be the most biologically plausible.

Another alternative class of Lambda-coalescent is the Durrett-Schweinsberg coalescent (Durrett and Schweinsberg  2005). This model describes populations undergoing successive hard selective sweeps throughout the genome, and in particular, the hitchhiking effect of those sweeps on a fixed, neutral site. The sweeps are modeled as points in a Poisson process of fixed rate. During a sweep, some ancestral lineages carrying the neutral site of interest can escape the sweep by recombining, while those lineages which do not recombine will merge to a common ancestor which initiated the sweep. Between individual sweeps the population follows neutral Moran type dynamics. This class of model has recently been found to describe the genetic diversity in cod populations (Árnason et al.  2023). In general, the measure Λ associated with this class of coalescents takes the form of $\text{Λ}=\delta_{0}+{\text{Λ}}_{0}$ where $\delta_{0}$ is an atom at 0 responsible for Kingman-like mergers between sweeps and ${\text{Λ}}_{0}$ is a finite measure on [0,1] without an atom at 0, which drives multiple mergers due to selective sweeps. This model is not directly applicable to pathogen populations, primarily due to cross-over recombination being less frequent in bacterial and viral pathogens. An adaptation of this model to bacterial pathogens may be possible but is outside of the scope of this study.

In infectious disease epidemiology, it is often important to consider the possibility of an overdispersed offspring distribution due to superspreading dynamics, and this is typically done using a negative-binomial offspring distribution (Lloyd-Smith et al.  2005; Grassly and Fraser  2008; Didelot et al.  2017). The coalescent framework can be used to represent genealogies under such arbitrary offspring distributions (Li et al.  2017; Fraser and Li  2017). Superspreading is likely to create detectable multifurcations but the parameters of the offspring distribution cannot directly be recovered from the parameters of a Lambda-coalescent model (Hoscheit and Pybus  2019). Alternative formulation using birth-death models instead of the coalescent framework is also popular for pathogen phylogenies (Stadler  2009, 2010; Stadler et al.  2013). This could make the link between offspring distribution and multifurcation more readily available, but would on the other hand require to specify a sampling model with good accuracy (Volz and Frost  2014).

Future studies are needed to investigate what type of Lambda-coalescents best describe pathogen dynamics. The approach presented here is an approximate, albeit explicit approach to Bayesian inference of multiple merger genealogies. It has limitations: for instance, it is not appropriate for studying genealogies spanning geological timescales, as was demonstrated by worsening bias as the number of substitutions per site becomes high (Fig. 6). It remains an open problem how to perform inference under Lambda-coalescents in the fully Bayesian setting, incorporating uncertainty about the phylogeny and relying on the phylogenetic likelihood. Applying some aspects of the parametrization and construction of multiple merger genealogies presented in this work in such a setting should be straightforward. However, we anticipate that the parametrization presented here may not be computationally efficient in this case.

Finally, there is the question of extending the approach presented here to, for example, joint estimation of varying effective population size (Ho and Shapiro  2011). This is relevant because the past effective population size is often a quantity of interest, but it is also important from the perspective of statistical robustness. If population growth is not accounted for, it can be misidentified as a Lambda-coalescent as was previously noted (Menardo et al.  2021). Likewise, geographical structuring of the pathogen population could confuse a model that assumes an unstructured population, as demonstrated by recent work on the spatial Lambda-Fleming-Viot model (Veber and Wakolbinger  2015; Biswas et al.  2021). In order to extend our model, for example to the case of a non-constant population size, there are separate questions that have to be answered. Firstly, the impact of varying effective population size on the genealogy depends on the forwards-in-time model. Therefore, it is first necessary to decide which Lambda-coalescent is the correct one to use for a given scenario. Secondly, adding a non-parametric model for the effective population size will increase the complexity of the inference problem. More efficient MCMC schemes, or other inference tools, would therefore need to be investigated. This might include non-reversible samplers with boundary conditions such as (Bierkens et al.  2023) or Hamiltonian Monte-Carlo methods (Dinh et al.  2017). Note that some phenomena might be indistinguishable from multiple mergers, such as population structure. For example, a sufficiently fast expansion of a subpopulation that shares identity by descent (Helekal et al.  2022) will likely lead to multiple mergers in the genealogy.

## SUPPLEMENTARY MATERIAL

Supplementary files are available on the Systematic Biology website.

## FUNDING

This work was supported by the UK Engineering and Physical Sciences Research Council (EPSRC) [grant EP/S022244/1 to D.H.] for the EPSRC Centre for Doctoral Training in Mathematics for Real-World Systems II; EPSRC Research [grant EP/V049208/1 to J.K.]; and the National Institute for Health and Care Research (NIHR) Health Protection Research Unit in Genomics and Enabling Data [NIHR200892 to X.D.].

## Supplementary Material

syaf003_suppl_Supplementary_Materials
