# Supplementary material for: Inference of Multiple Mergers while Dating a Pathogen Phylogeny
Source: Syst Biol. 2025 Jan 18;74(6):897–916. doi: 10.1093/sysbio/syaf003 (PMC12712333; doi:10.1093/sysbio/syaf003)
Supplement: syaf003_suppl_Supplementary_Materials [file syaf003_suppl_supplementary_materials.pdf]

Inference of multiple mergers while dating a pathogen phylogeny Supplementary Material

David Helekal, Jere Koskela, Xavier Didelot

MCMC scheme

We use four types of moves to sample parameters characterising Y. These moves can be categorised into three families. The first family consists of moves covering transitions that update the position within a full dimensional orthant including the boundary. The moves within the first family update branch lengths, and therefore merger sizes and internal node heights. The second family consists of moves for proposing transitions between full dimensional orthants. The moves within the second family update the root position and branching order within polytomies. The third family consists of a single move that updates the parameters of the observation model and genealogy model. The scheme consists of a single sweep through all three families, selecting one move from each family uniformly at random.

Orthant interior move

The orthant interior move is a random walk Metropolis (RWM) move restricted to updating a subset of the vector $\boldsymbol{\chi}$ corresponding to those coordinates which are currently not restricted to the boundary, and thus a part of a multiple merger

$$k_{\mathrm{RWM}}(\chi_i' \mid \chi_i, q_i)_{\boldsymbol{\sigma}} = \mathbb{1}_0(q_i)\delta_0 + |\mathcal{N}|(\chi_i' - \chi_i; \sigma_i), \tag{S1}$$

where $|\mathcal{N}|(\cdot;\sigma)$ denotes the density of the modulus of a normal random variable with variance σ^2. As expected for RWM the proposal ratio equals to one.

Orthant boundary move

The orthant boundary move is responsible for transitioning between binary topologies and multiple mergers. To do so the move needs to propose transitions between the boundary and orthant interior. The main challenge with designing this move is that due to the structure of the likelihood of the extended Beta-coalescent there is a relatively sharp ridge between a 2-merger and a 3-merger when the number of active lineages is large, i.e. $b \gg 3$. Therefore the move must be able to propose transitions that move several coordinates of χ to 0 or back at once. The first step consists of sampling an internal node index $i \in \boldsymbol{I} \setminus \{2n-1\}$ at random with probability

$$P\left[i = x \mid \boldsymbol{\chi}\right] \propto w_1(\chi(x)) \tag{S2}$$

If the coordinate is allocated to the interior (therefore to the slab component of the base measure), i.e. if $q(i) = 0, \chi(i) > 0$ then the move begins by proposing $\chi'(i) = 0$. Denoting the parent node above $j = \mathrm{pa}(i)$ the moves continues upwards and proposing to shrink $\chi(j)$ to 0 with probability $w_2(\chi(x))$ as long as it is accessible to this move, i.e. $q(\mathrm{pa}(j)) = 0$ and $j \neq 2n-1$. This process repeats until a coordinate fails to shrink either due to it not being accessible or due to the coin flip failing.
If the coordinate initially selected is allocated to the boundary then there are two options. The move either expands that coordinate, proposing $\chi'(i) > 0$. Alternatively the move expands that coordinate and then attempts to expand the coordinate above it if it is allocated to the boundary. If the coordinate above it is allocated to the boundary it is expanded and this procedure repeats, terminating when either the root is reached or a non-zero coordinate is reached. The expanded coordinates are sampled from a proposal distribution d, i.e. $\chi' \sim d$. Whether the move expands coordinates recursively or not is decided uniformly at random. Denote the sequence of nodes that have had their corresponding

coordinates modified by $v_1, v_2, ..., v_m$, where v_1 is the first node modified by the move. If the initial coordinate chosen was allocated to the interior the proposal ratio is equal to

$$
\begin{aligned}
a(\boldsymbol{\chi}', \boldsymbol{\chi}) = & \frac{w_1(\chi'(v_1)) \sum_{j \in \boldsymbol{I} \setminus \{2n-1\}} w_1(\chi(j))}{w_1(\chi(v_1)) \sum_{j \in \boldsymbol{I} \setminus \{2n-1\}} w_1(\chi'(j))} d(\chi(v_1))^{1-2q(v_1)} \\
& \times \left(\mathbb{1}_{x \geq 2}(n) \prod_{v_j : 2 \leq j \leq n} \left(\frac{d(\chi(v_j))}{w_2(\chi(v_j))} \right)^{1-2q(v_1)} + \mathbb{1}_1(n) \right) \\
& \times \left[\frac{1}{2} \left(1 + \mathbb{1}_1(n)\right) \right]^{1-2q(v_1)} \\
& \times \left(\frac{1}{P_s} \right)^{1-2q(v_1)}
\end{aligned}
\tag{S3}
$$

Note that $1 - 2q(v_1) = 1$ if the move is shrinking coordinates towards the boundary and -1 if it is expanding coordinates. Therefore it determines the direction of the move. The first term corresponds to the probability of selecting the same starting node, the second term corresponds to the likelihood of the coordinate transformation for subsequent nodes, the third accounts for the possibility of the reverse move being chosen, and the fourth term corresponds accounts for the stopping probability of the recursion, P_s which is equal to

$$
P_s = \begin{cases} 1 - w_2(\text{pa}(v_n)) & \text{if pa}(v_n) \text{ is accessible} \\ 1 \quad \text{otherwise} \end{cases}
\tag{S4}
$$

Crucially setting $w_2(\chi)$ to be equal to the density of $d(\chi)$ leads to the second term cancelling to one. In practice we use

$$
\begin{aligned}
d(x) &= \frac{\sqrt{2}}{b\sqrt{\pi}} \exp\left\{ -\frac{x^2}{2b^2} \right\} \mathbb{1}_{x \geq 0} \\
w_2(x) = w_1(x) &= \exp\left\{ -\frac{x^2}{2a^2} \right\}
\end{aligned}
\tag{S5}
$$

Setting $a = b = \sqrt{2/\pi}$ leads to $d(x) = w_1(x) = w_2(x)$.

Root NNI move

The root NNI move updates the root position, and is used if the root position is considered unknown. It is a version of the nearest neighbour interchange (NNI) commonly used in phylogenetic inference.This moves first proceeds in selecting one of the child nodes of the root as a pivot. The move then moves the root to one of the descendant edges of the pivot. The pivot is chosen with equal probability from both root descendant nodes at random with the exception of two special cases. If one of the descendant nodes is a tip node the other descendant of the root is selected as a pivot. This is because NNI move is undefined for a tip node selected as a pivot. The other special case is if one of the root descendants has the branch above it collapsed as a part of a multiple merger but the other does not. In this case the descendant with the edge collapsed is chosen as the pivot. This is to prevent the move from wasting computational effort as this way the node with edge collapsed stays adjacent to the root. Otherwise it may become adjacent to an edge that cannot support a multiple merger leading to 0 likelihood. With the pivot selected the branch to move the root to is sampled with equal probability from two edges that descend from the pivot. We denote the pivot by $p \in \boldsymbol{C_\tau}$ and its probability mass function under the topology $\boldsymbol{\tau}$

$$
P_{\boldsymbol{\tau}}[p = i] = \begin{cases} \frac{1}{2}, & \text{if } (c_l > n) \wedge (c_r > n) \wedge (q(c_l) = q(c_r)) \\ \mathbb{1}_{c_l}(i), & \text{if } (c_r \leq n) \vee ((q(c_l) = 1) \wedge (q(c_r) = 0)) \\ \mathbb{1}_{c_r}(i), & \text{if } (c_l \leq n) \vee ((q(c_r) = 1) \wedge (q(c_l) = 0)) \end{cases}
\tag{S6}
$$

With p fixed, sample j from the two descendants of p uniformly at random. Based on this generate a new rooted tree topology $\boldsymbol{\tau}'$, which is the same as $\boldsymbol{\tau}$ except where:

$$\mathrm{pa}_{\boldsymbol{\tau}'}(i) = \begin{cases} p, & \text{if } i \in \boldsymbol{C}_{\boldsymbol{\tau}} \wedge i \neq p \\ 2n-2, & \text{if } i = j \\ \mathrm{pa}_{\boldsymbol{\tau}}(i), & \text{otherwise} \end{cases} \tag{S7}$$

The mutations above the affected nodes are then adjusted accordingly:

$$m_{\boldsymbol{\tau}'}(i) = \begin{cases} m_{\boldsymbol{\tau}}(c_r) + m_{\boldsymbol{\tau}}(c_l), & \text{if } i \in \boldsymbol{C}_{\boldsymbol{\tau}} \wedge i \neq p \\ \lfloor m_{\boldsymbol{\tau}}(j)s \rfloor, & \text{if } i = j \\ \lfloor m_{\boldsymbol{\tau}}(j)(1-s) \rfloor, & \text{if } i = p \\ \mathrm{pa}_{\boldsymbol{\tau}}(i), & \text{otherwise} \end{cases} \tag{S8}$$

For some arbitrarily chosen $s \in [0, 1]$.
The proposal ratio is

$$a(\boldsymbol{\tau}', \boldsymbol{\tau}) = \frac{P_{\boldsymbol{\tau}'}[p = i]}{P_{\boldsymbol{\tau}}[p = i]} \tag{S9}$$

Polytomy neighbour interchange move

The polytomy neighbour interchange move changes the binary topology within polytomies as this topology is randomly resolved (if at all) by the ML estimation program. It does so by first sampling a pivot node p from all nodes for which the edge above contains 0 mutations. With the pivot selected a node j is chosen uniformly at random from all the descendants of p that are adjacent to the polytomy. That is from all descendant nodes such that the parent of that node is 0 mutations away from p. Denote this set of nodes $A_{\boldsymbol{\tau}}$. Next a node k is selected from all nodes in the outgroup relative to p such that the distance of the parent of those nodes to the parent of p is 0 mutations. Denote this set of nodes $B_{\boldsymbol{\tau}}$. Based on this generate a new rooted tree topology $\boldsymbol{\tau}'$, which is the same as $\boldsymbol{\tau}$ except where:

$$\mathrm{pa}_{\boldsymbol{\tau}'}(i) = \begin{cases} \mathrm{pa}_{\boldsymbol{\tau}}(j), & \text{if } i = k \\ \mathrm{pa}_{\boldsymbol{\tau}}(k), & \text{if } i = j \\ \mathrm{pa}_{\boldsymbol{\tau}}(i), & \text{otherwise} \end{cases} \tag{S10}$$

The reverse move consists of selecting the same pivot and then the corresponding descendants. Denote the sibling of the pivot p with s_p. The proposal ratio is

$$a(\boldsymbol{\tau}', \boldsymbol{\tau}) = \frac{|A_{\boldsymbol{\tau}'}|^{-1}|B_{\boldsymbol{\tau}'}|^{-1} + (1 - \mathbb{1}_{s_p}(k))(|A_{\boldsymbol{\tau}'}| + 1)^{-1}(|B_{\boldsymbol{\tau}'}| - 1))^{-1}}{|A_{\boldsymbol{\tau}}|^{-1}|B_{\boldsymbol{\tau}}|^{-1} + (1 - \mathbb{1}_{s_p}(k))(|A_{\boldsymbol{\tau}}| + 1)^{-1}(|B_{\boldsymbol{\tau}}| - 1))^{-1}} \tag{S11}$$

Tuning the sampler

In order to tune the sampler a preconditioning matrix of individual parameter variances is estimated, along with a scale factor multiplying this matrix. The RWM moves updating the parameters and node height variables are then scaled accordingly. This is done in a sequence of six steps. Let T denote the thinning factor. The first two steps consists of running the sampler with orthant boundary moves disabled. The first step consists of burn-in for $100 \times T$ iterations. The second step then estimates the χ preconditioning matrix for $100 \times T$ iterations. For the following steps all moves are enabled. The third step consists of burn-in for $100 \times T$ steps. The fourth step estimates the preconditioning matrix for the parameter moves for $100 \times T$ steps. The fifth and sixth steps estimate the step scaling for $50 \times T$ steps. All previous iterations are then discarded.

	Avg. Bias	RMSE	90% Quantile Range	Statistics
LSD2	-0.217	0.232	0.287	Relative Branch Count
	0.511	0.714	1.60	Relative Tree Height
	0.438	0.637	1.54	Relative Tree Length
TreeTime	-0.113	0.142	0.274	Relative Branch Count
	112	2562	2.08	Relative Tree Height
	117	2751	1.59	Relative Tree Length
Ours	0.0160	0.0298	0.0772	Relative Branch Count
	0.000653	0.125	0.401	Relative Tree Height
	-0.00703	0.0776	0.251	Relative Tree Length

Table S1: Comparison of branch count, tree height and tree length estimated by LSD2, TreeTime and our method for the simulation benchmark under the extended Beta-coalescent.

	Beta Simulation	Extended Beta Simulation
LSD2	19.8	20.3
TreeTime	41.1	3884
Ours	11.6	10.6

Table S2: Comparison of branch score distances for LSD2, TreeTime and our method for both simulation benchmarks. Note that in the case of our method we compute the expected posterior branch score distance for each run.

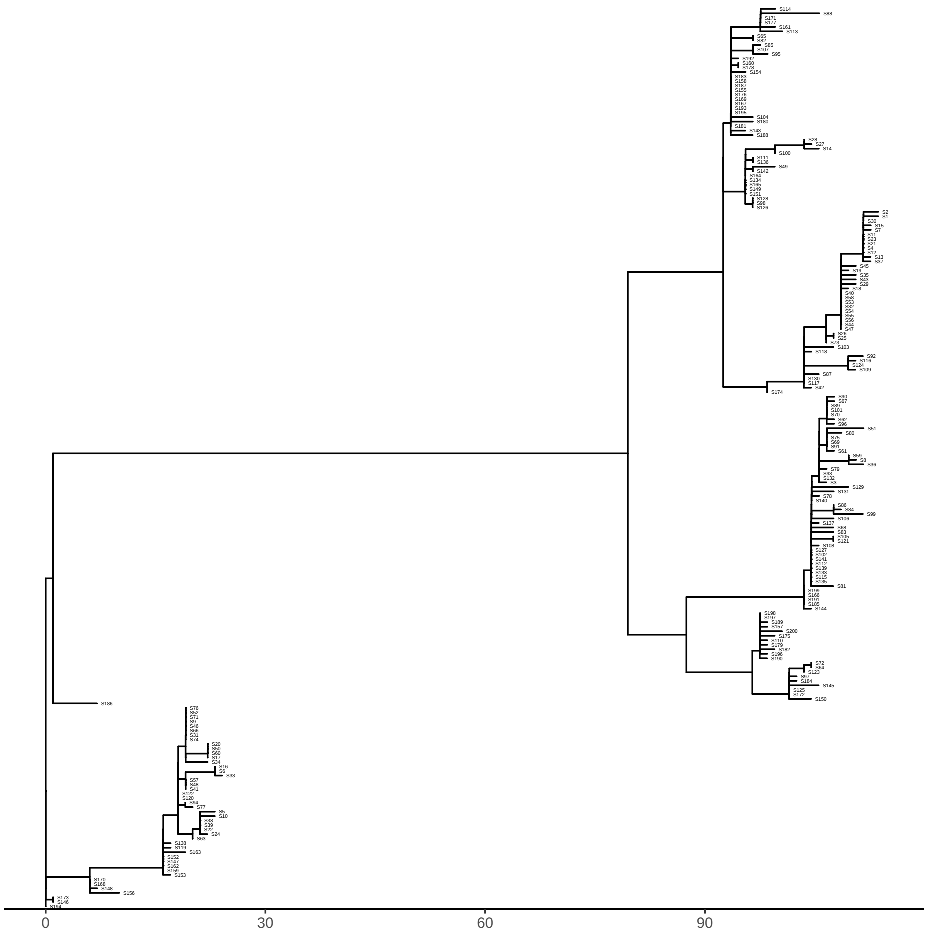

Figure S1: Input phylogeny for the Beta-coalescent example.

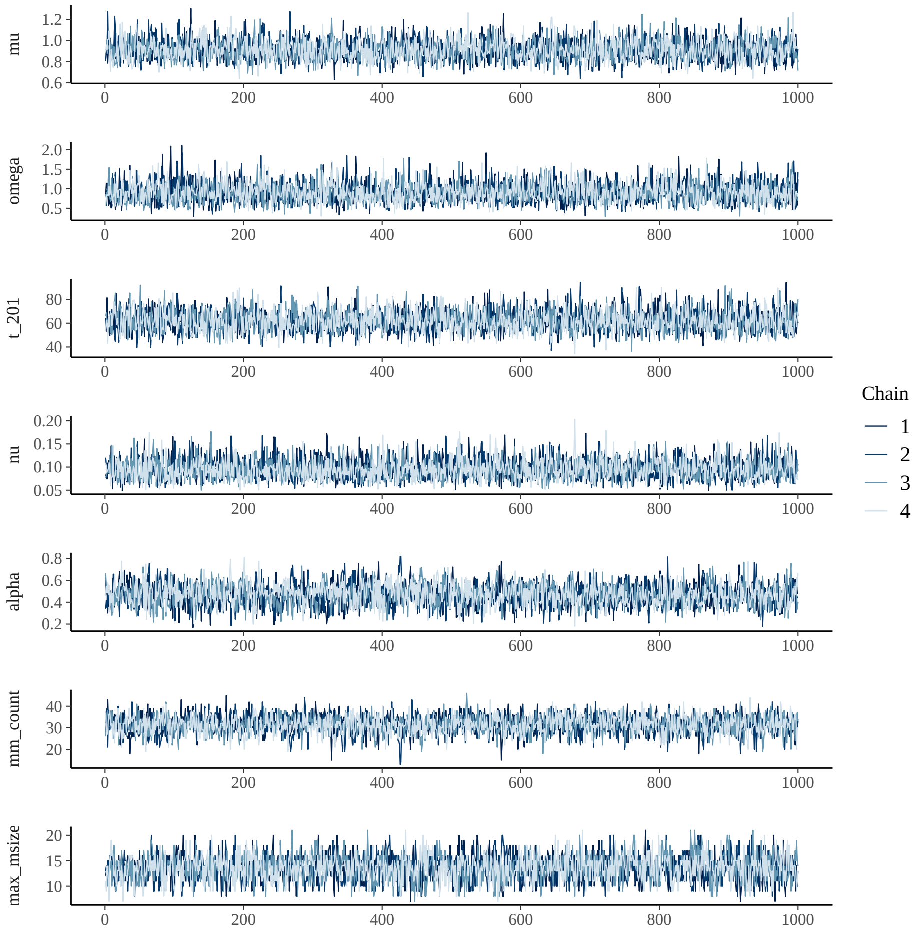

Figure S2: Parameter and diagnostic quantity traces for the Beta-coalescent example.

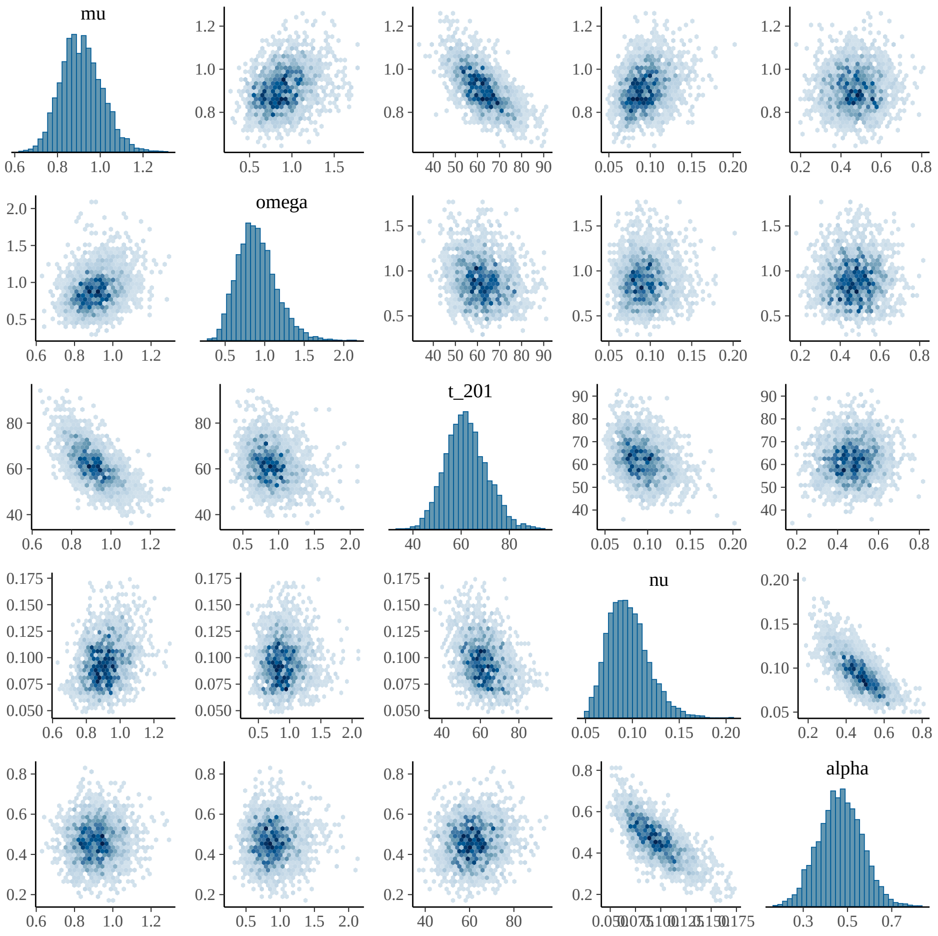

Figure S3: Parameter posterior distributions for the Beta-coalescent example.

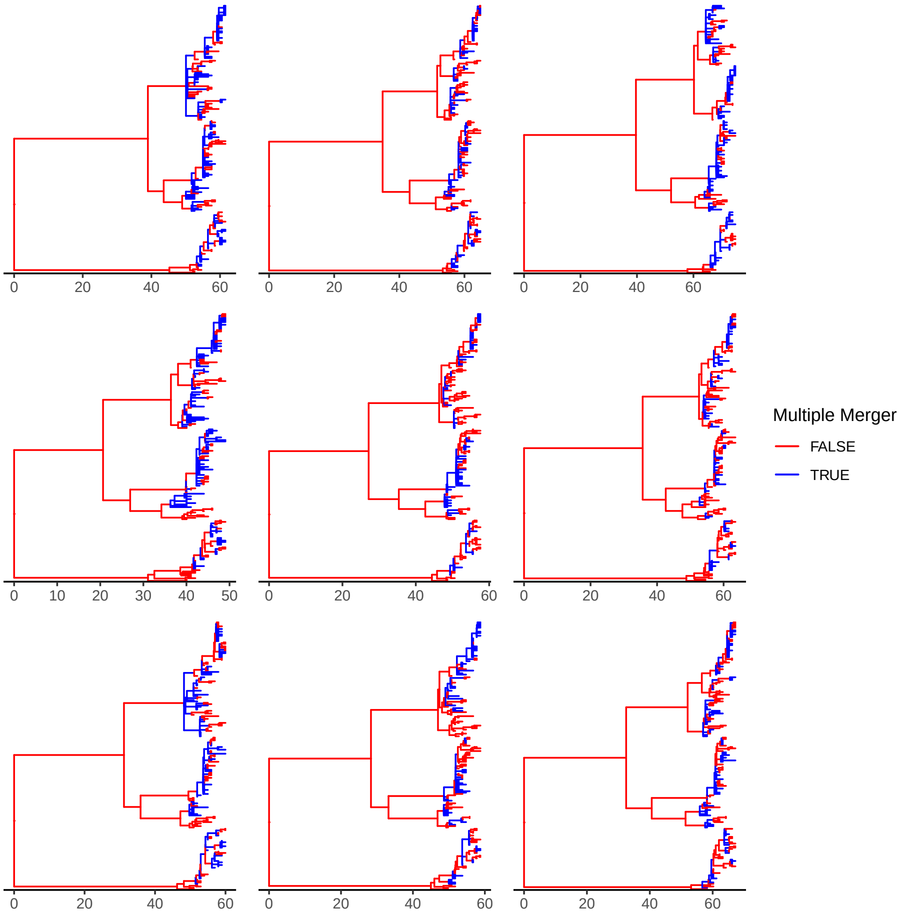

Figure S4: Nine realisations of the underlying genealogy sampled from the posterior estimated for the Beta-coalescent example. Branches are colored according to presence of multiple merger directly above.

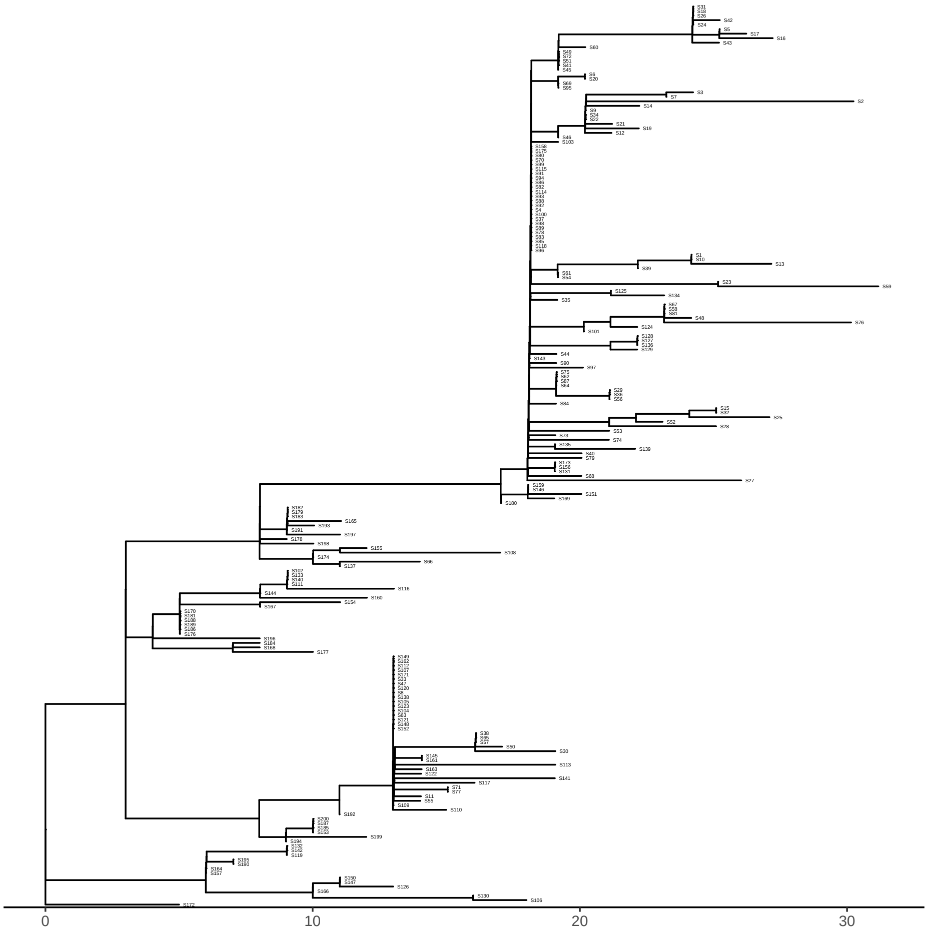

Figure S5: Input phylogeny for the extended Beta-coalescent example.

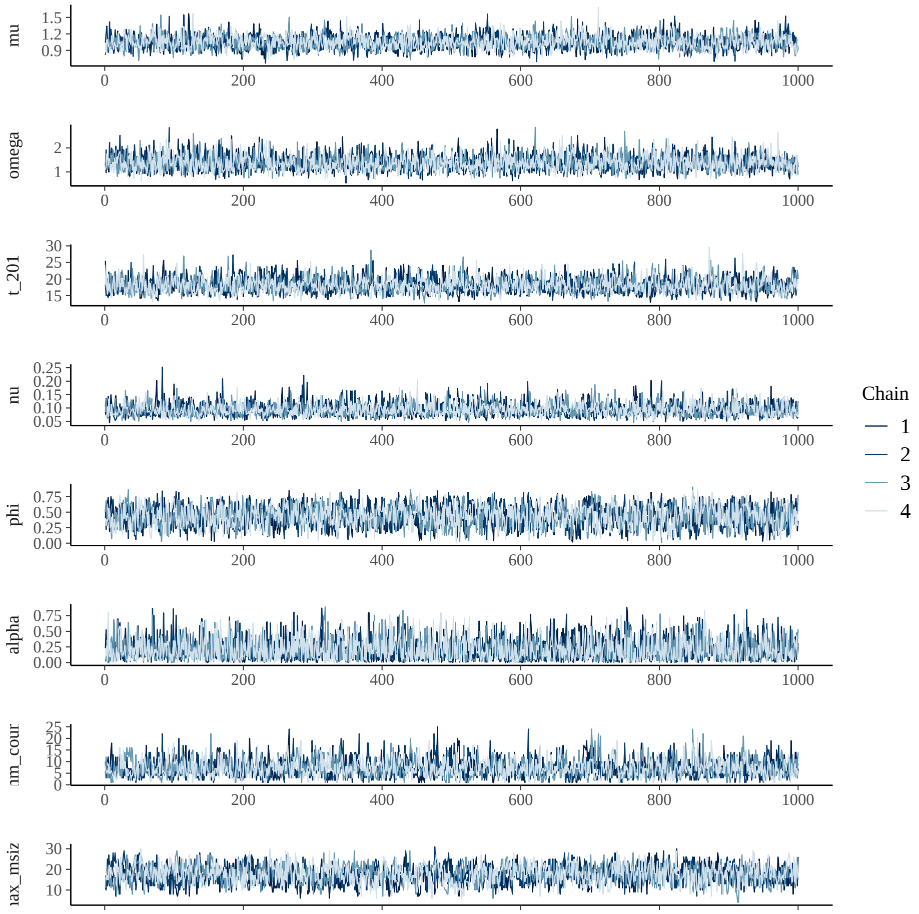

Figure S6: Parameter and diagnostic quantity traces for the extended Beta-coalescent example.

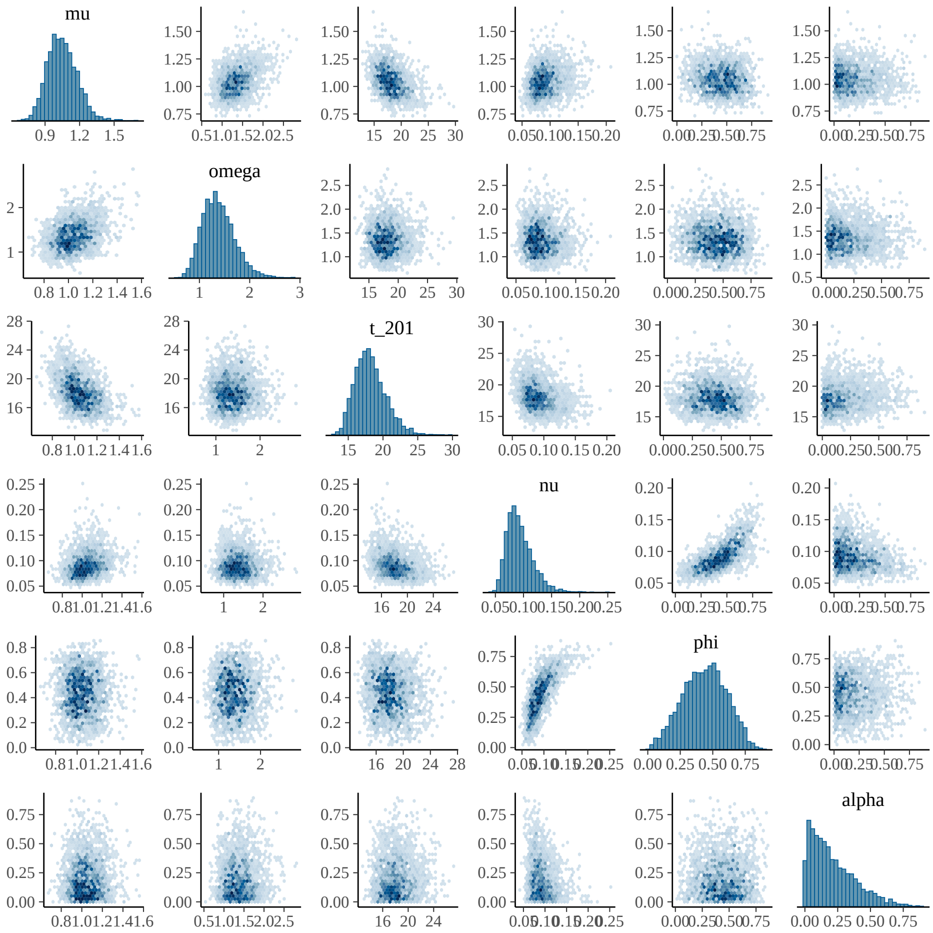

Figure S7: Parameter posterior distributions for the extended Beta-coalescent example.

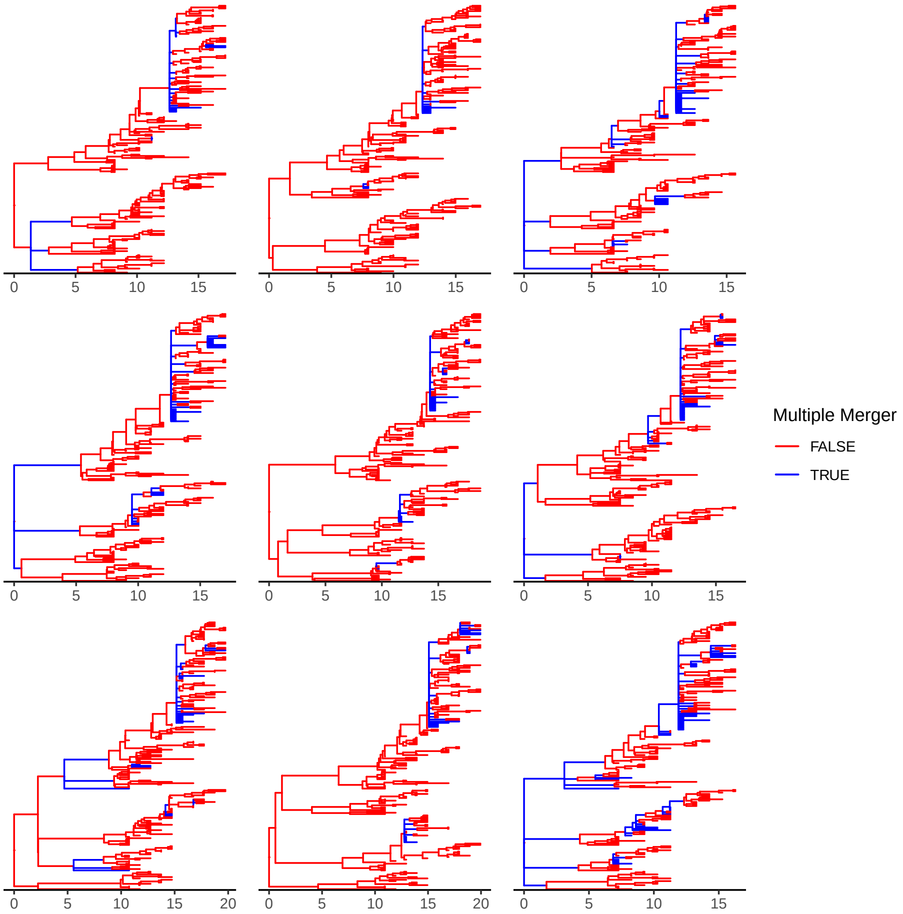

Figure S8: Nine realisations of the underlying genealogy sampled from the posterior estimated for the extended Beta-coalescent example. Branches are colored according to presence of multiple merger directly above.

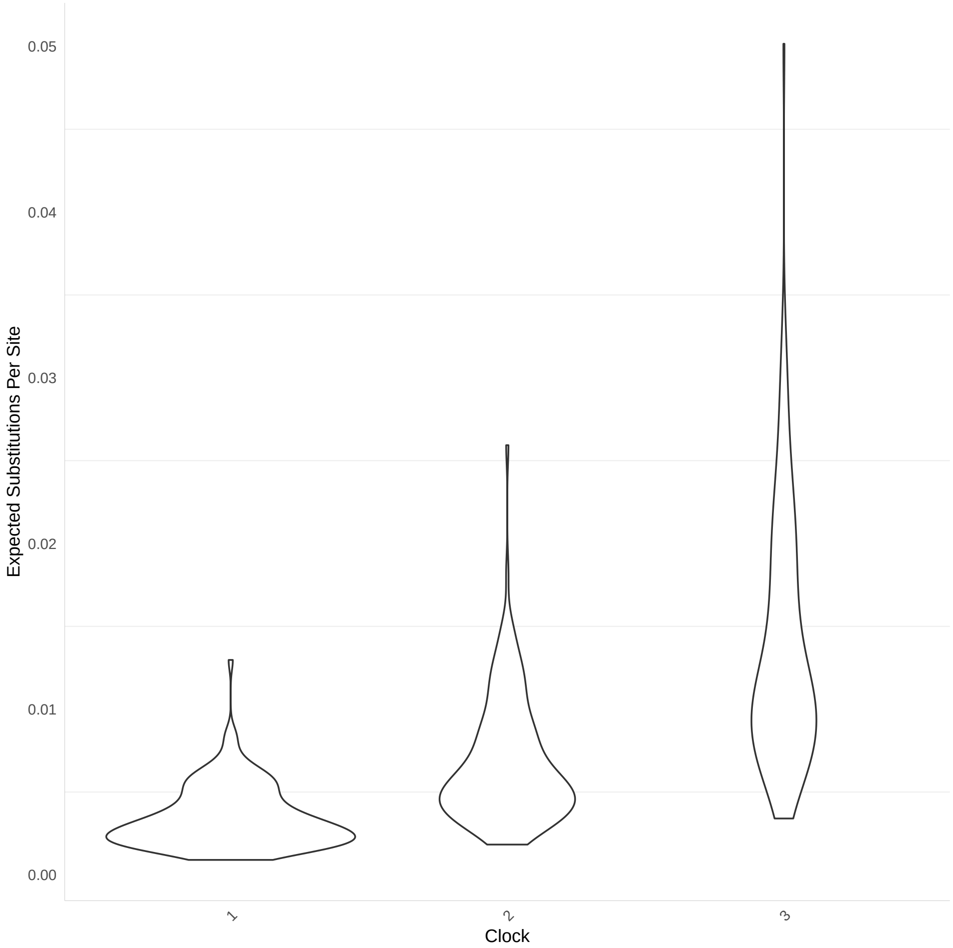

Figure S9: Expected number of substitutions for simulated sequences used for the analysis under the Beta-coalescent.

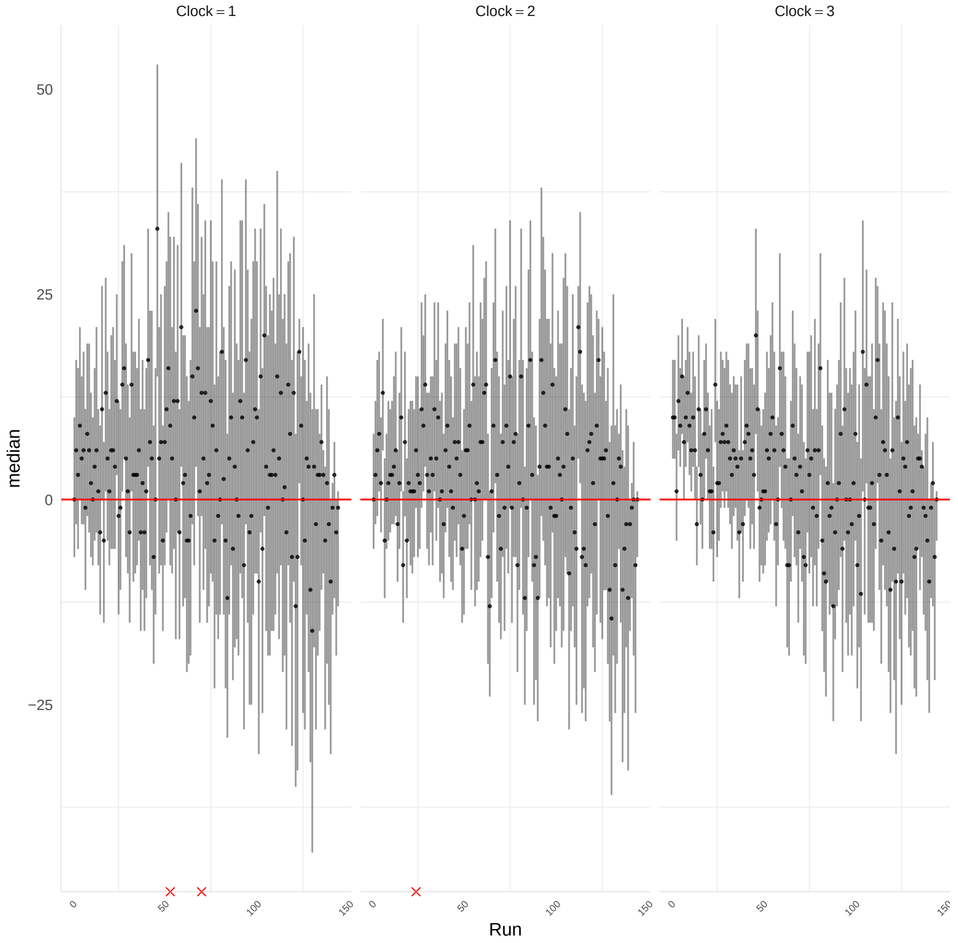

Figure S10: Number of nodes in posterior samples minus the true number of nodes in the simulated genealogy for the analysis under the Beta-coalescent. Red crosses indicate runs that indicated unsatisfactory mixing.

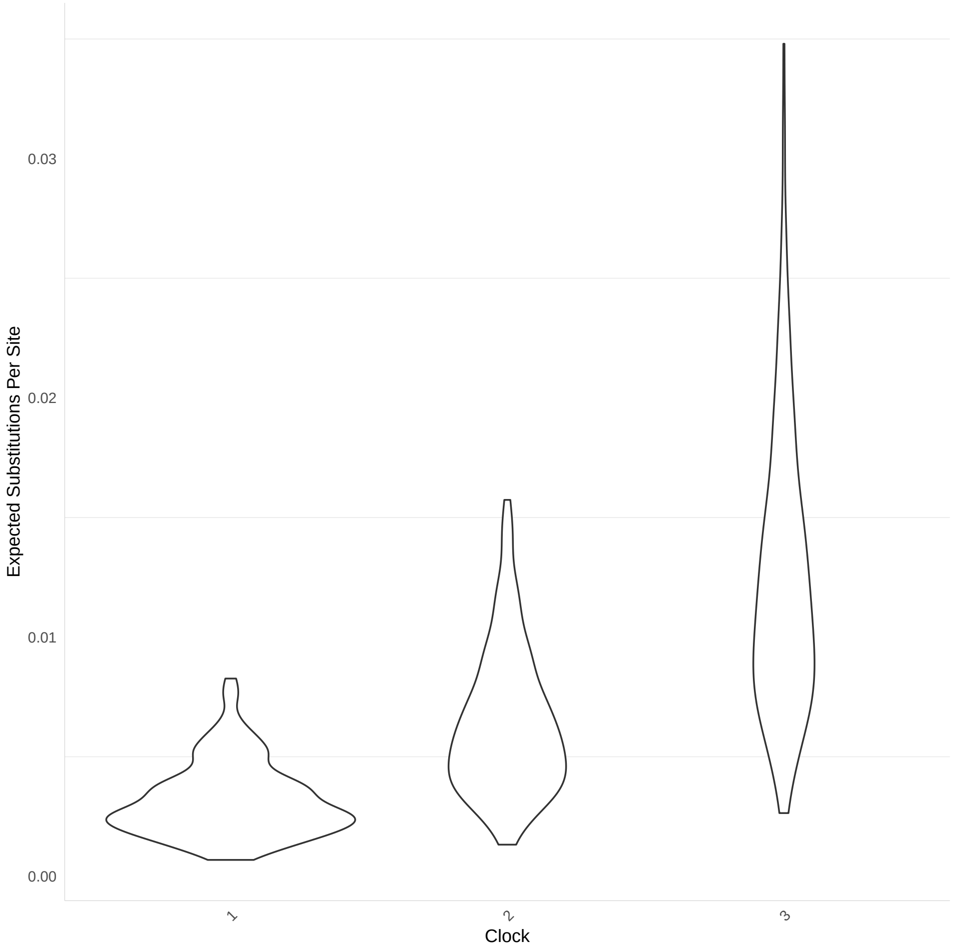

Figure S11: Expected number of substitutions for simulated sequences used for the analysis under the extended Beta-coalescent. Red crosses indicate runs that indicated unsatisfactory mixing.

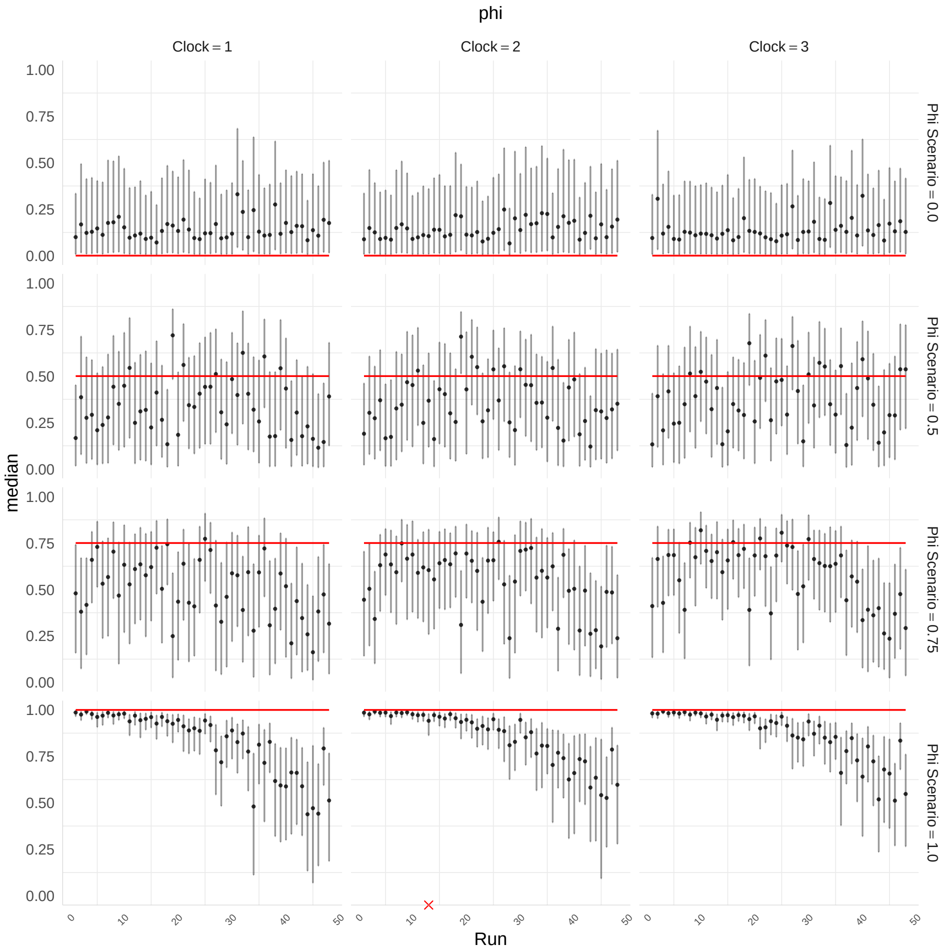

Figure S12: Posterior summaries for the ϕ parameter for the analysis under the extended Beta-coalescent. Red crosses indicate runs that indicated unsatisfactory mixing.

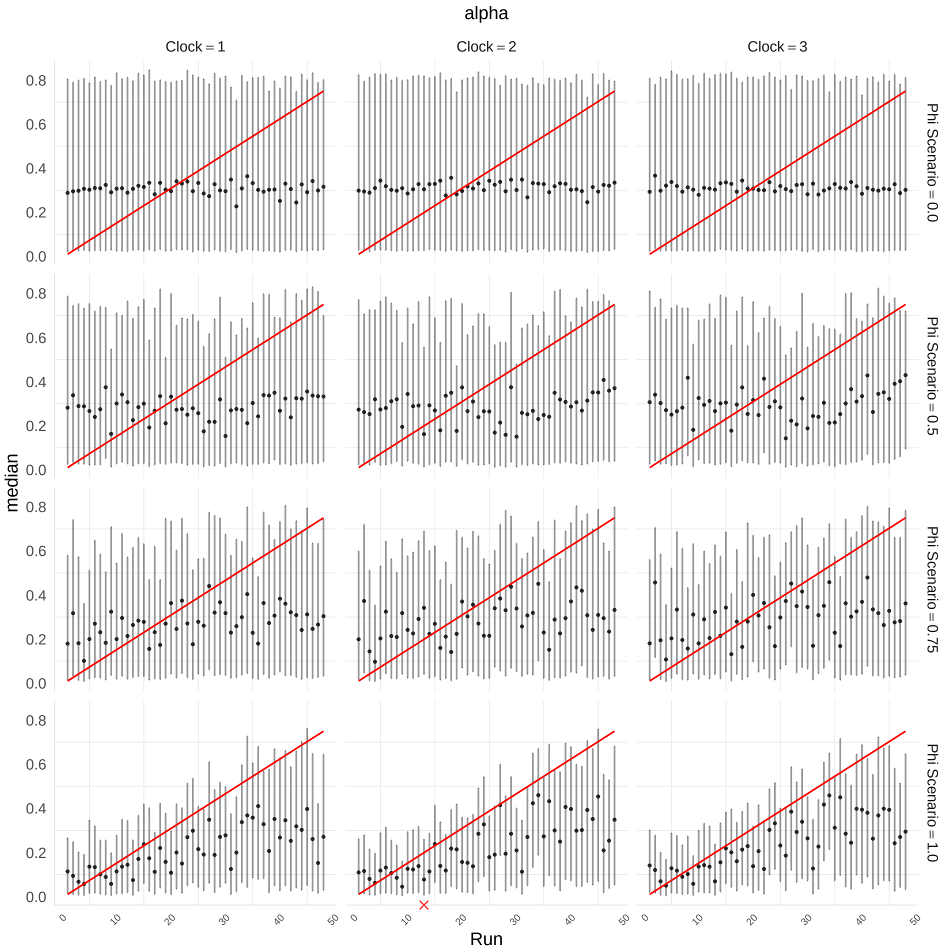

Figure S13: Posterior summaries for the α^* parameter for the analysis under the extended Beta-coalescent.

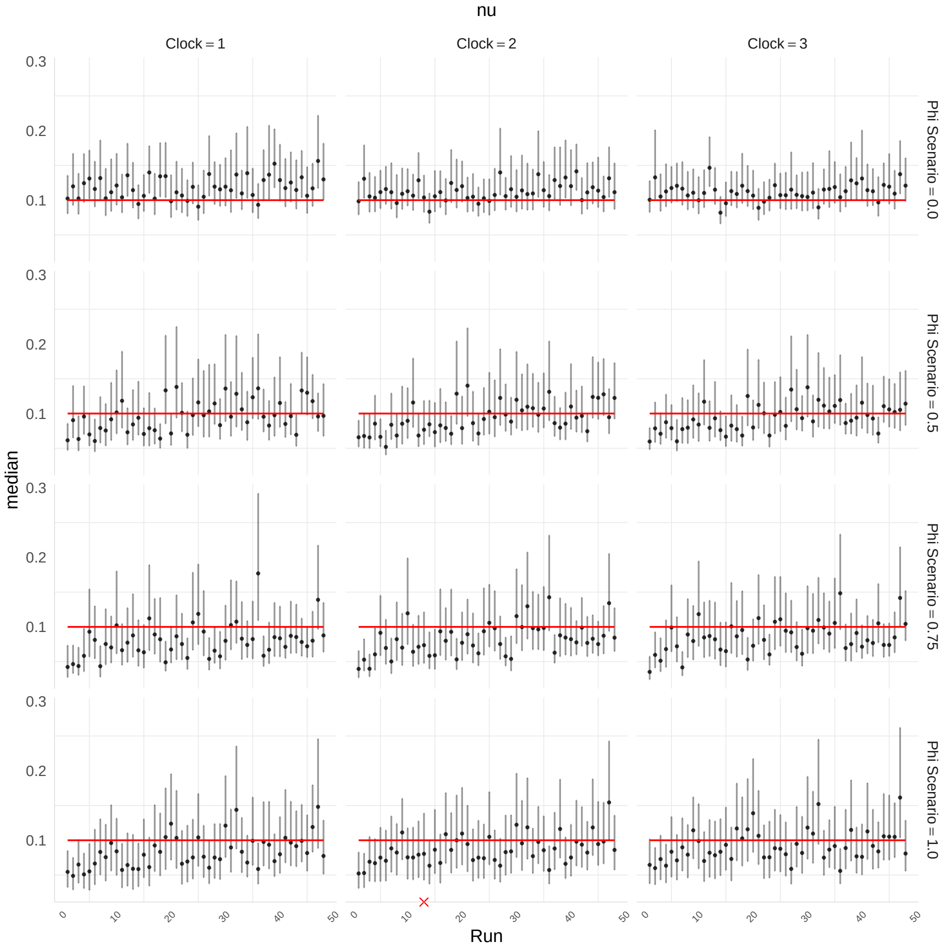

Figure S14: Posterior summaries for the ν parameter for the analysis under the extended Beta-coalescent. Red crosses indicate runs that indicated unsatisfactory mixing.

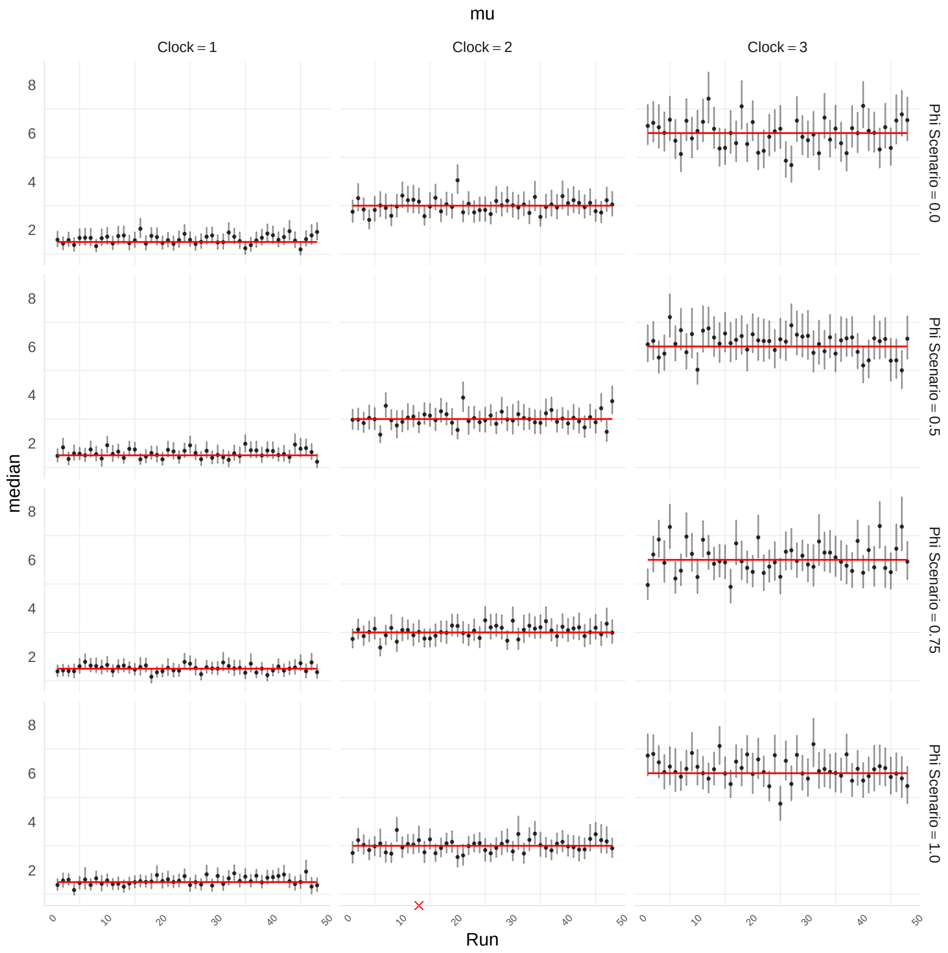

Figure S15: Posterior summaries for the μ parameter for the analysis under the extended Beta-coalescent. Red bold line indicates simulation value. Red crosses indicate runs that indicated unsatisfactory mixing.

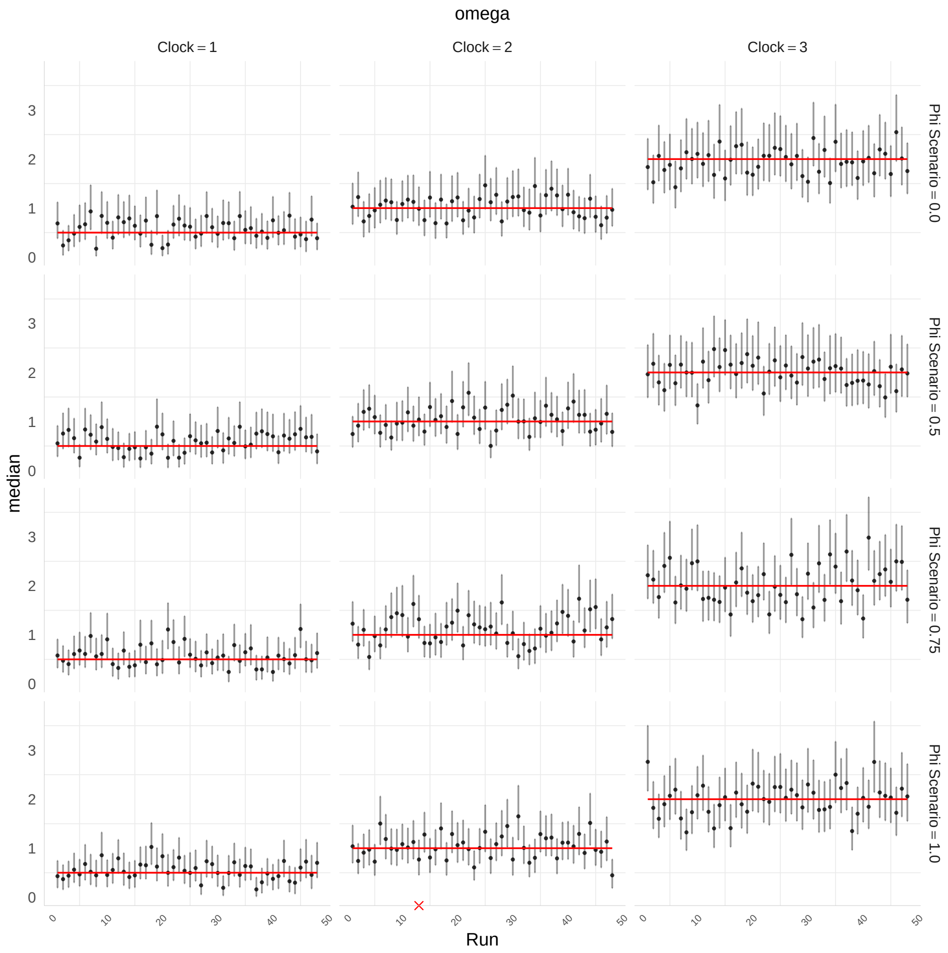

Figure S16: Posterior summaries for the ω parameter for the analysis under the extended Beta-coalescent. Red bold line indicates simulation value. Red crosses indicate runs that indicated unsatisfactory mixing.

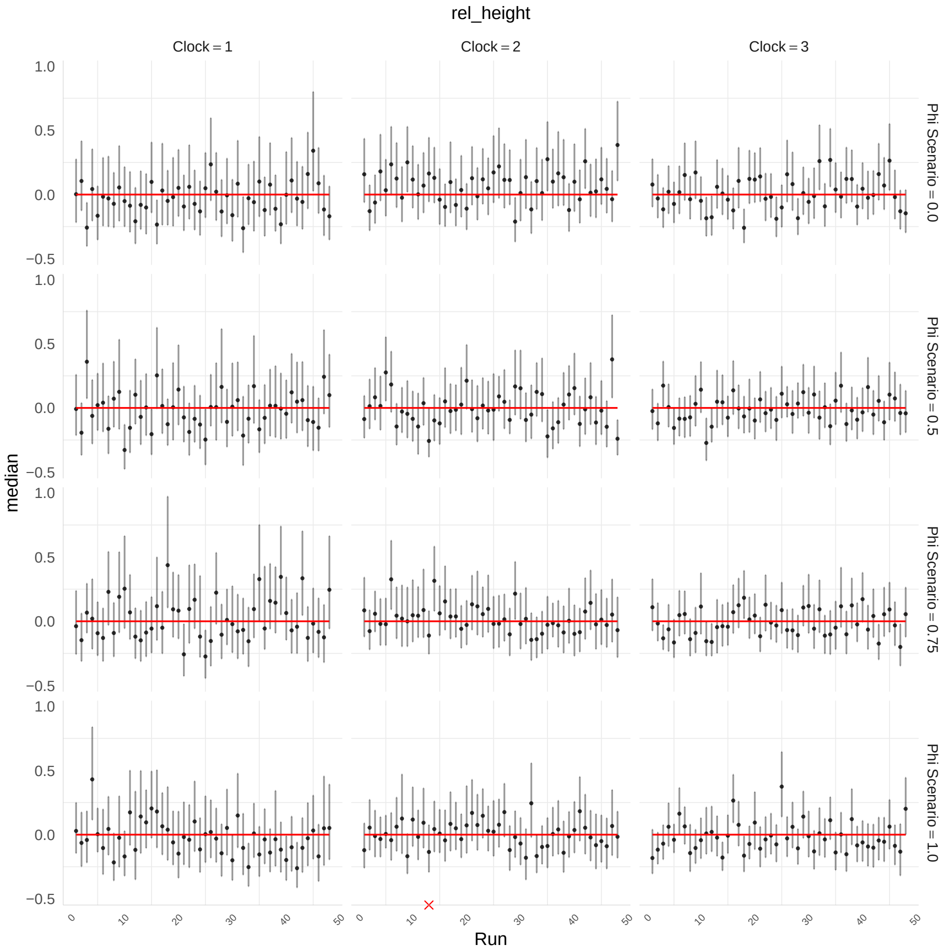

Figure S17: Posterior summary of relative tree heights for the analysis under the extended Beta-coalescent. Red crosses indicate runs that indicated unsatisfactory mixing.

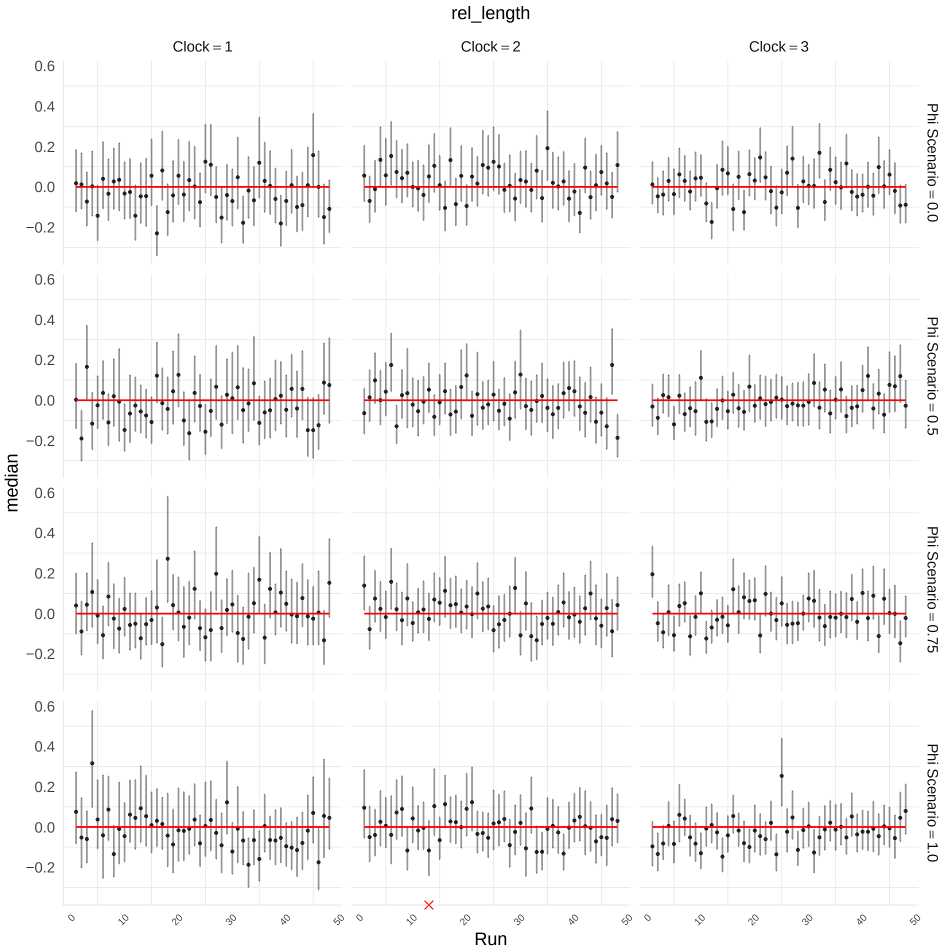

Figure S18: Posterior summary of relative tree lengths for the analysis under the extended Beta-coalescent. Red crosses indicate runs that indicated unsatisfactory mixing.

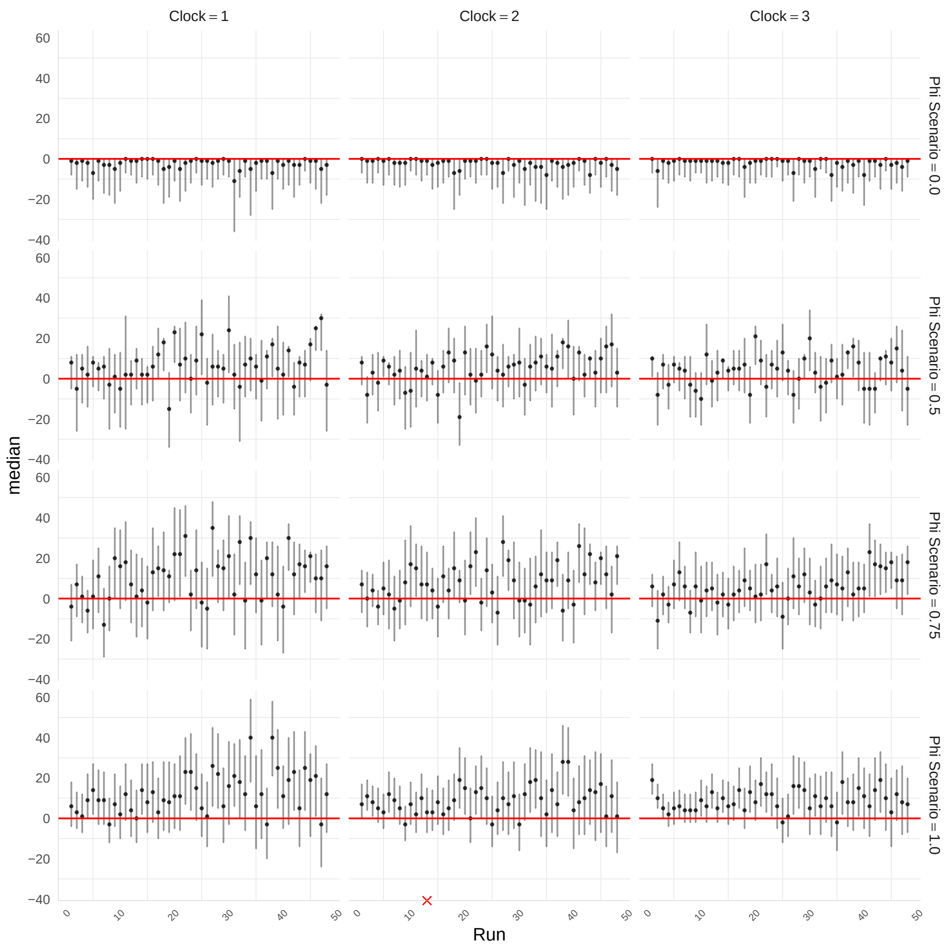

Figure S19: Number of nodes in the posterior samples minus the number of nodes in the simulated genealogy for the analysis under the extended Beta-coalescent. Red crosses indicate runs that indicated unsatisfactory mixing.

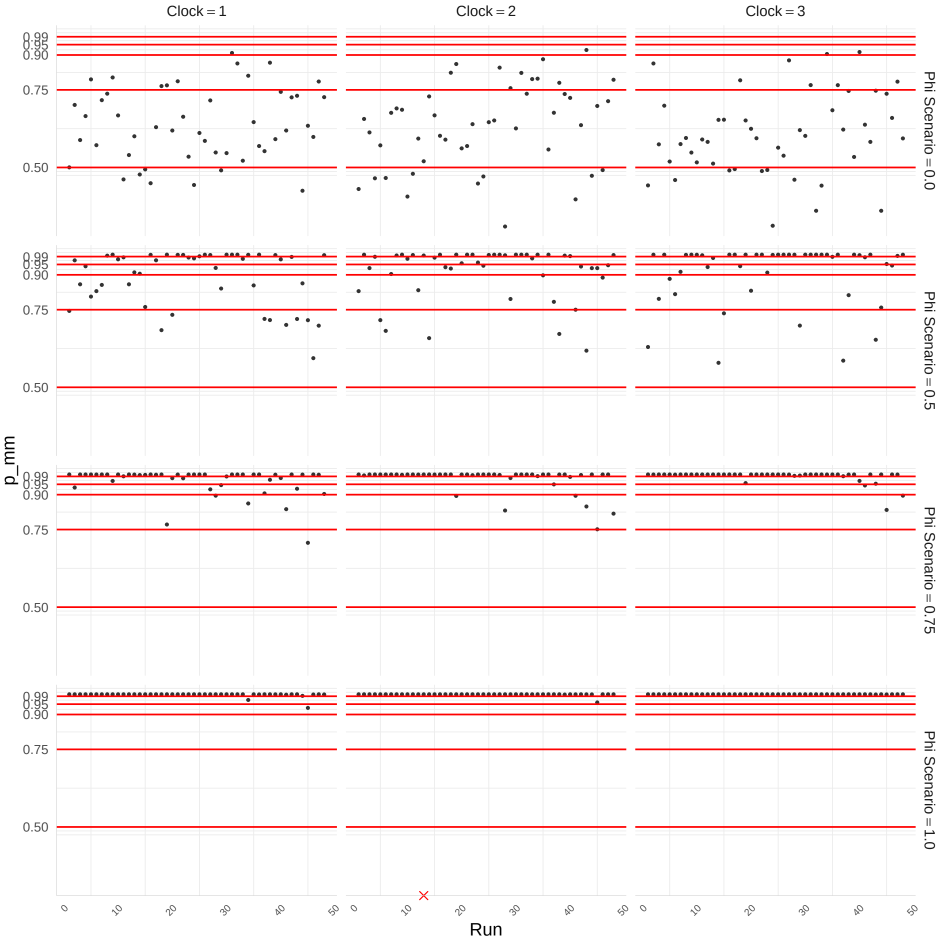

Figure S20: Estimated probabilities that a tree sampled from the posterior contains at least one multi-merger event for the analysis under the extended Beta-coalescent. Red lines indicate the 50%, 75%, 90%, 95%, and 99% thresholds. Red crosses indicate runs that indicated unsatisfactory mixing.

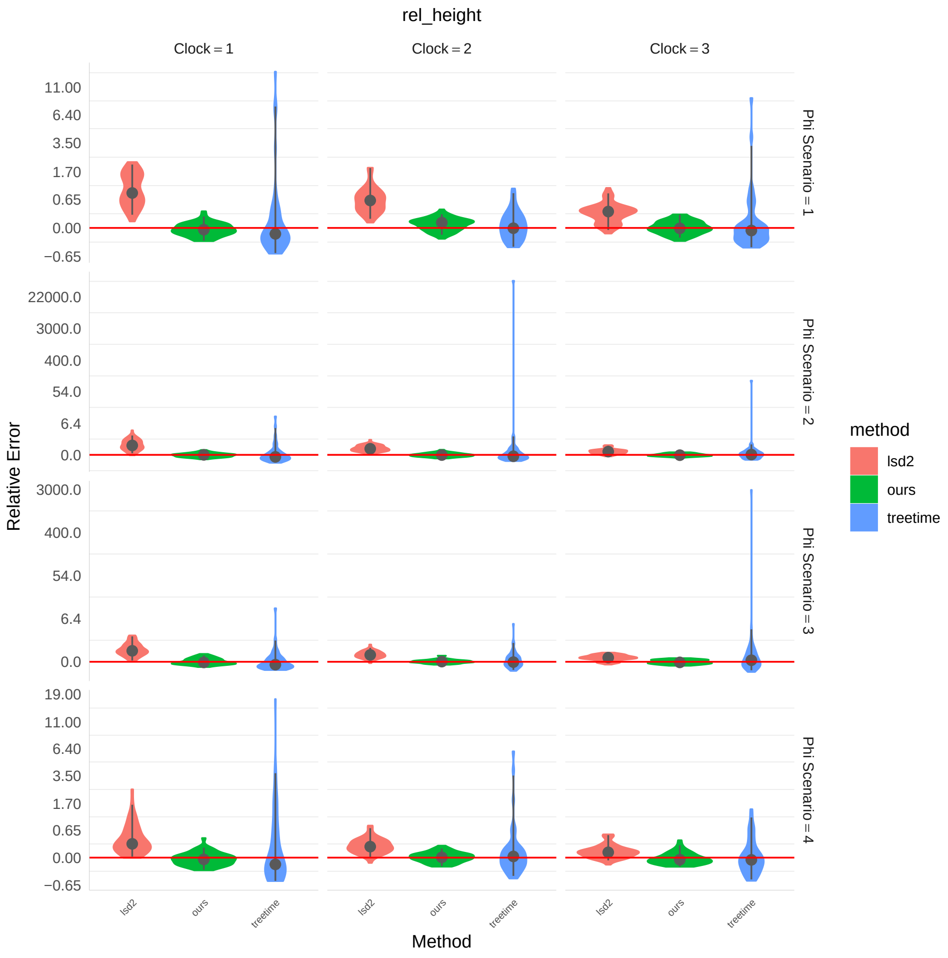

Figure S21: Tree heights estimated by LSD2, TreeTime and our method for the analysis under the extended Beta-coalescent.

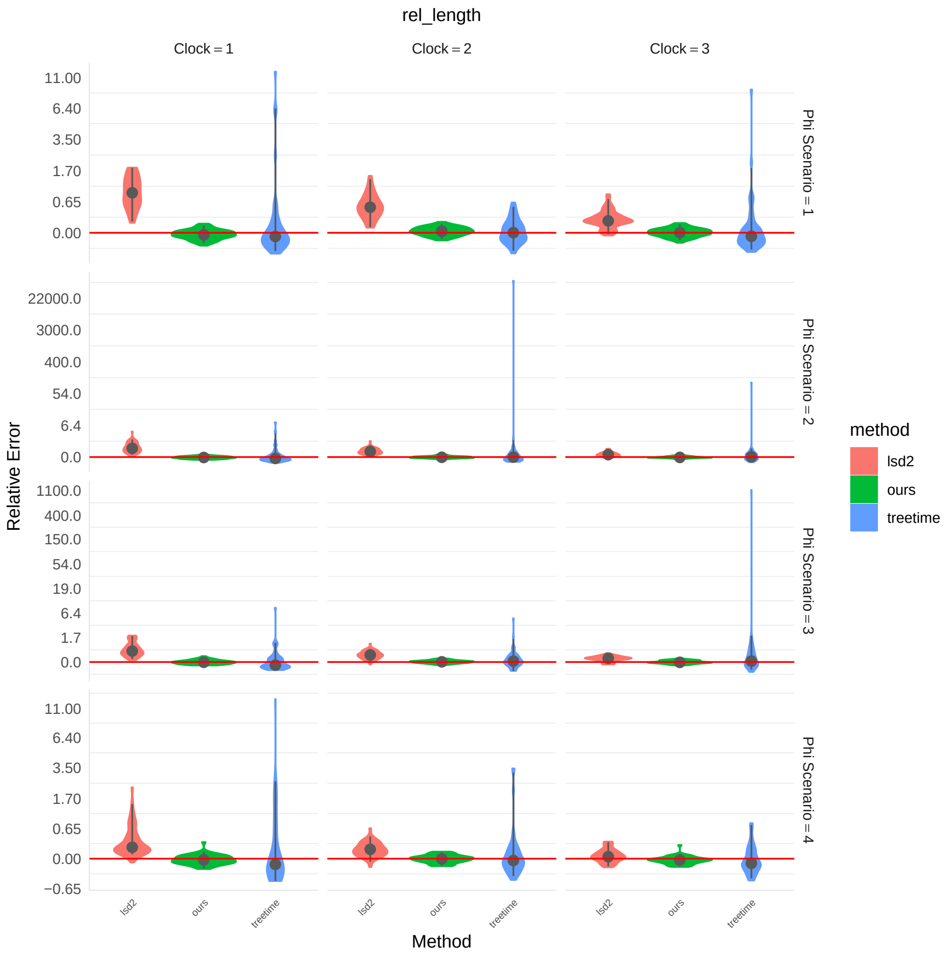

Figure S22: Tree heights estimated by LSD2, TreeTime and our method for the analysis under the extended Beta-coalescent.

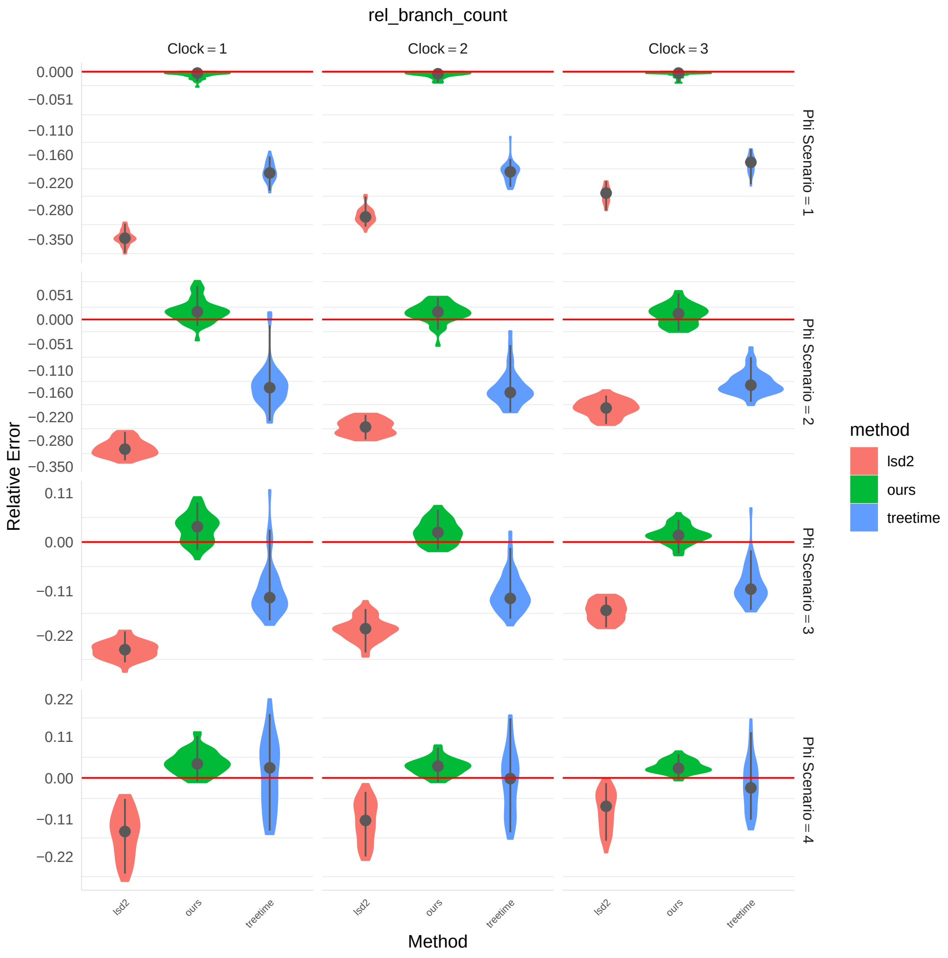

Figure S23: Tree heights estimated by LSD2, TreeTime and our method for the analysis under the extended Beta-coalescent.

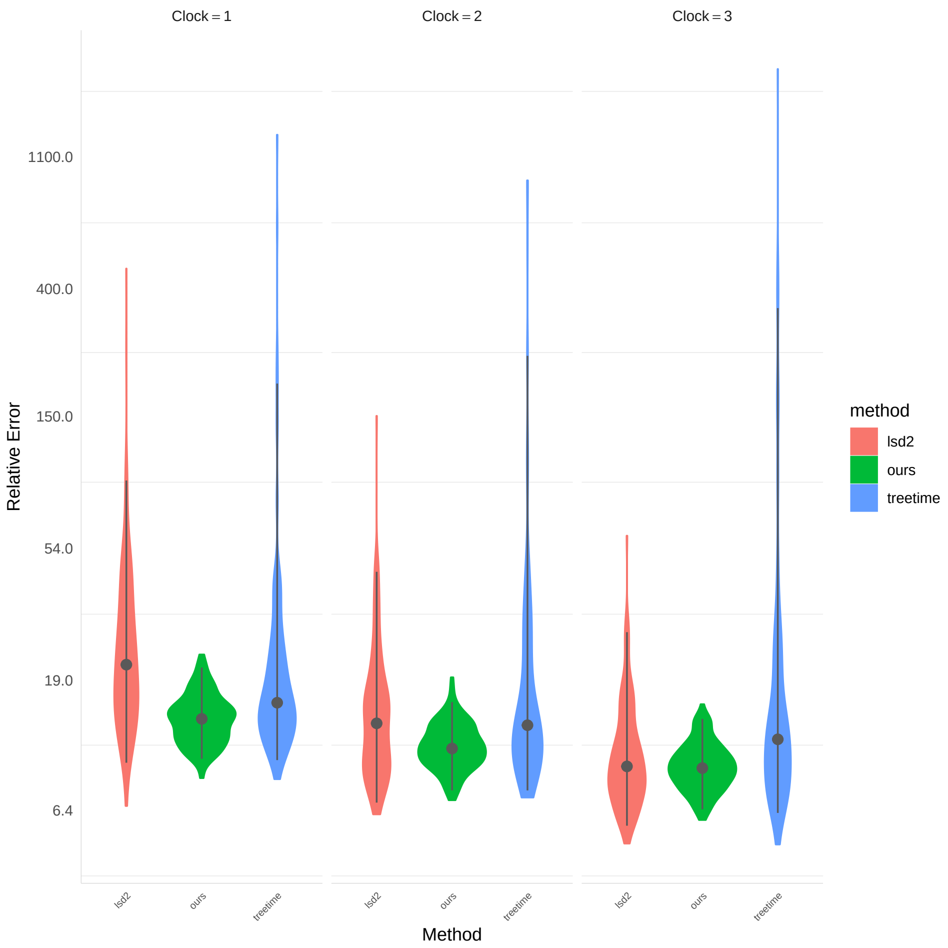

Figure S24: Branch score distances between time trees estimated by LSD2, TreeTime, and our method, and the true tree for the analysis under the Beta-coalescent. Note that for our method we compute the expected posterior branch score distance.

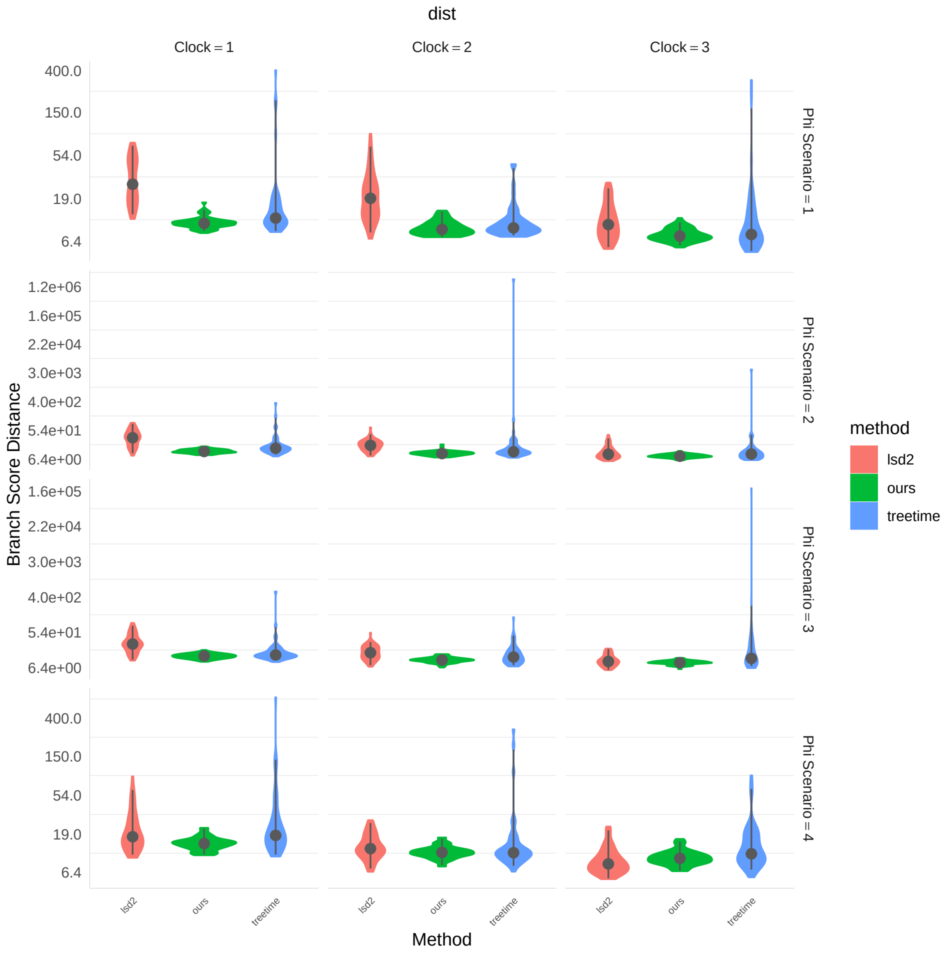

Figure S25: Branch score distances between time trees estimated by LSD2, TreeTime, and our method, and the true tree for the analysis under the extended Beta-coalescent. Note that for our method we compute the expected posterior branch score distance.

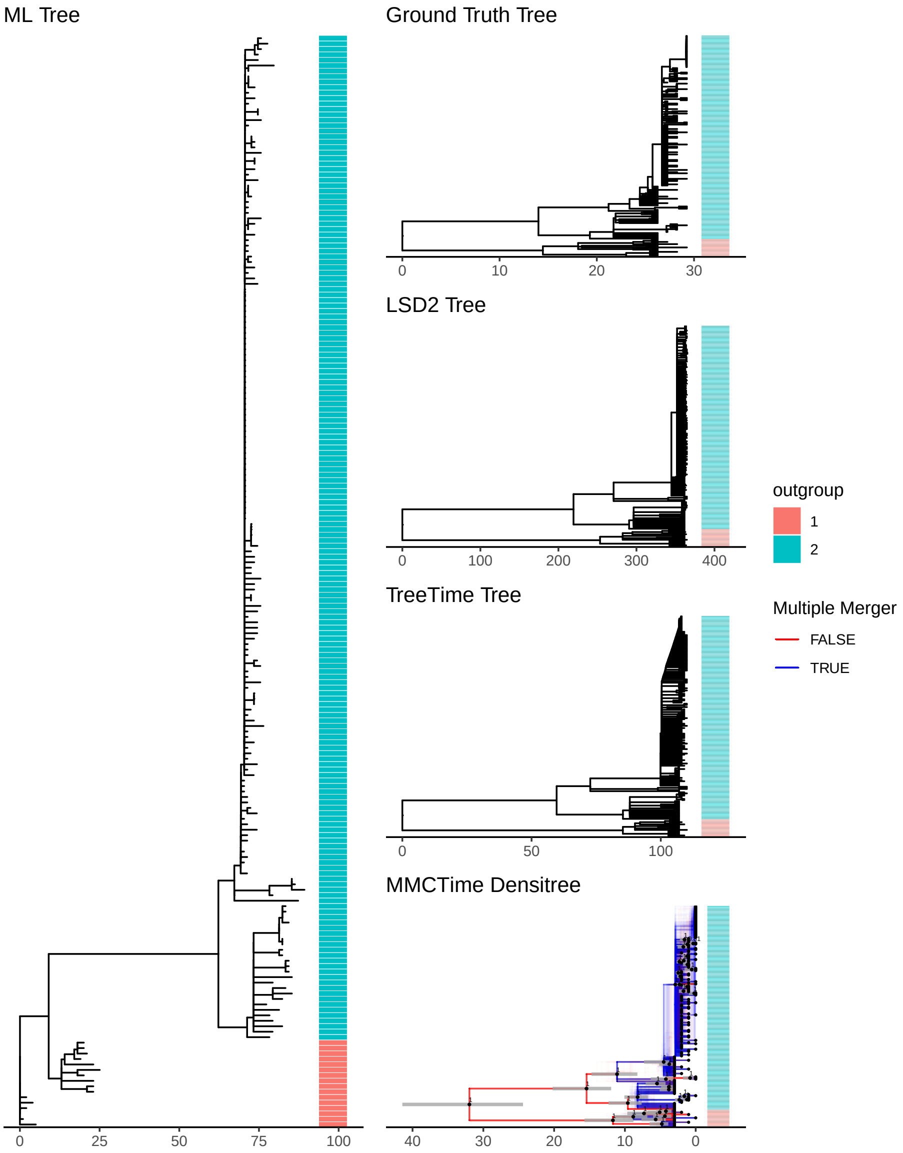

Figure S26: An example of Beta-coalescent simulation where both LSD2 and TreeTime had high relative errors.

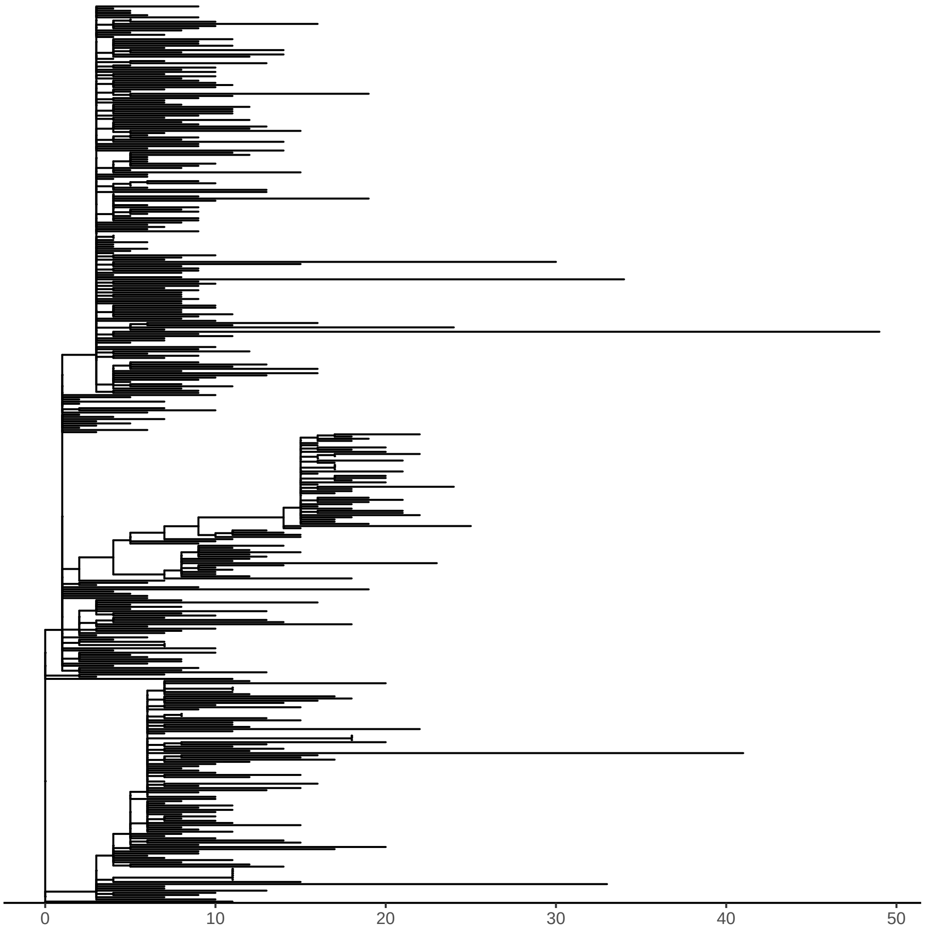

Figure S27: Input phylogeny for the *Vibrio cholerae* case study.

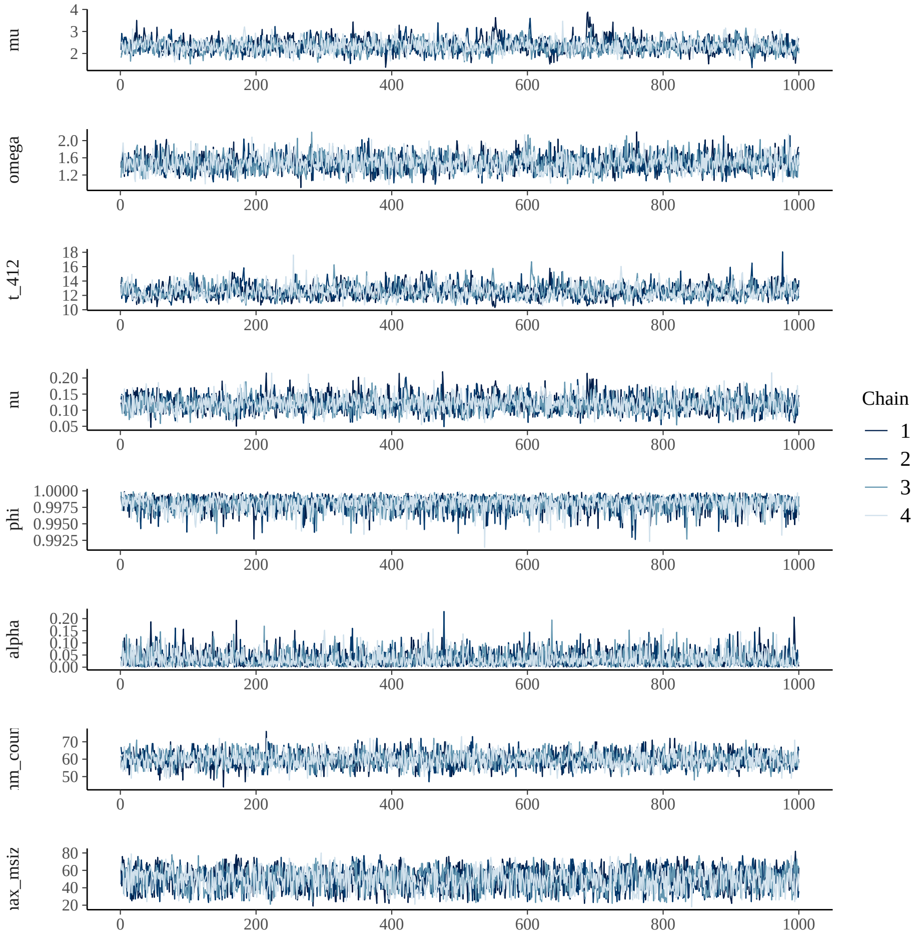

Figure S28: Parameter and diagnostic quantity traces for the analysis of the *Vibrio cholerae* dataset under the extended Beta-coalescent.

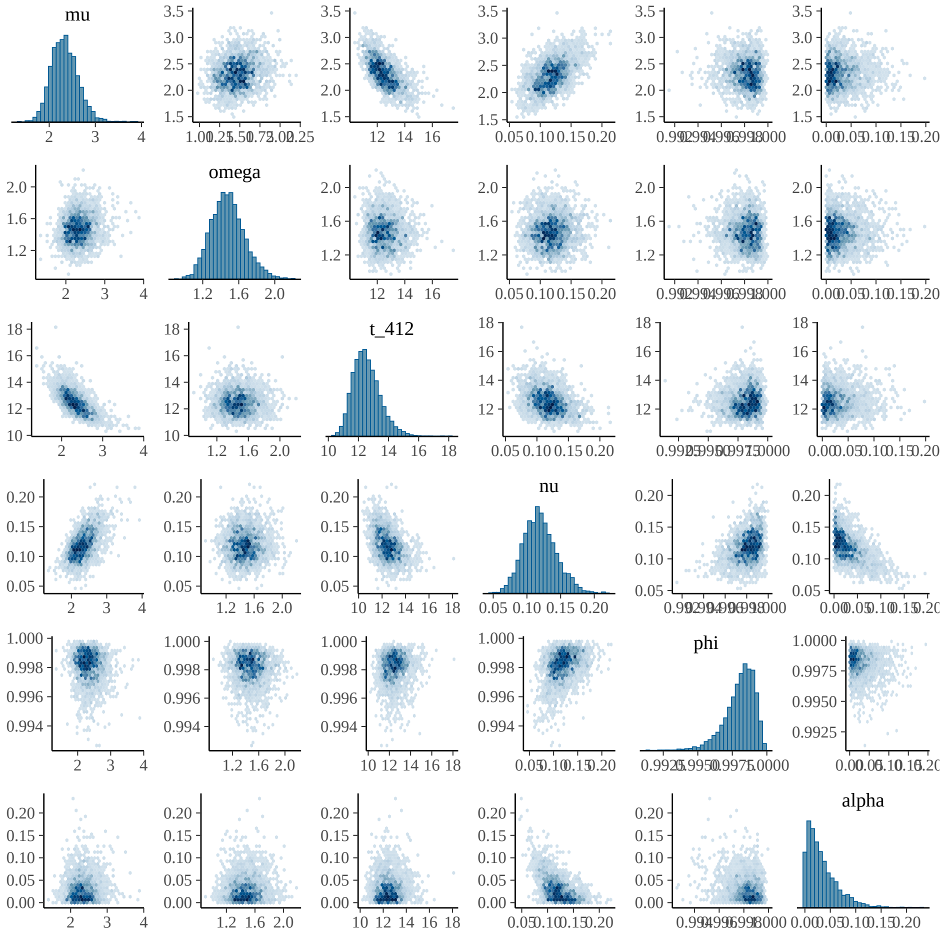

Figure S29: Estimated posterior parameter marginals for the analysis of the *Vibrio cholerae* dataset under extended Beta-coalescent.

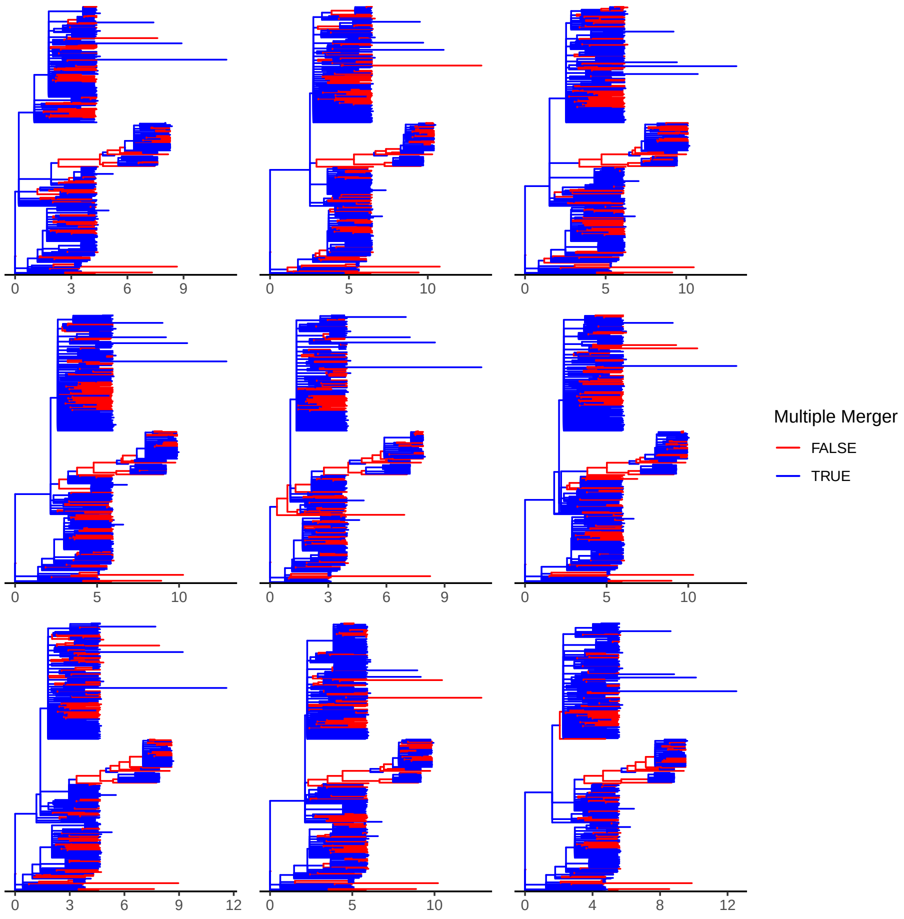

Figure S30: Nine posterior realisations of the underlying genealogy for the analysis of the *Vibrio cholerae* dataset under the extended Beta-coalescent.

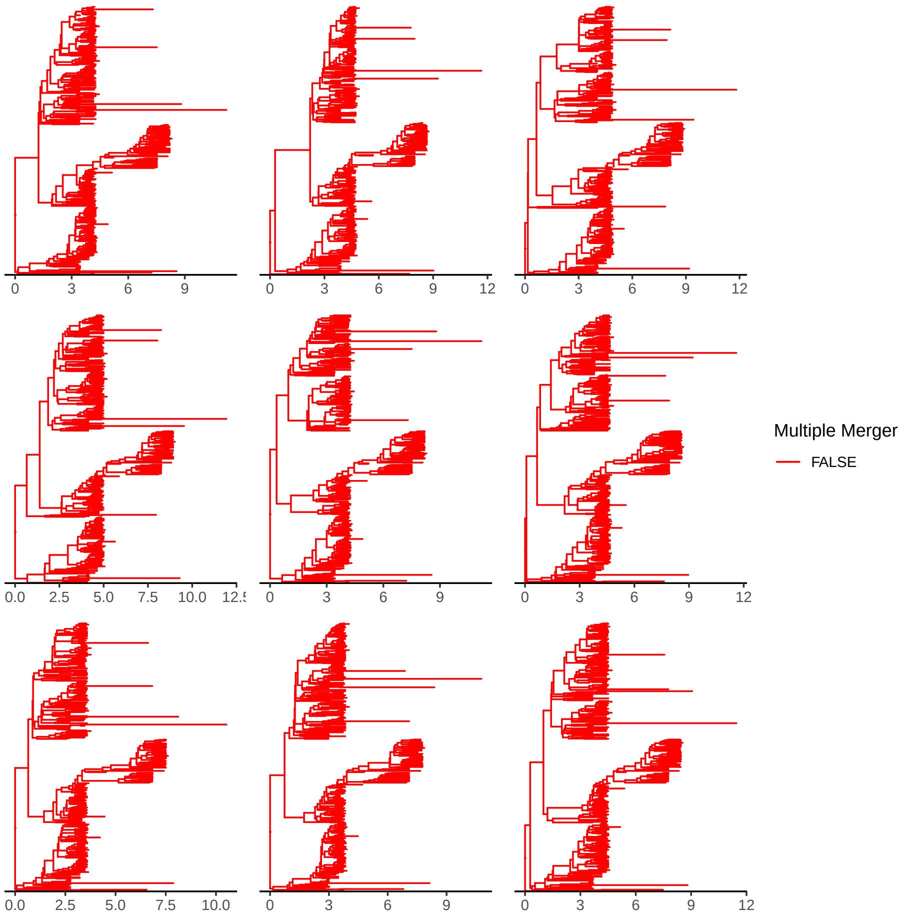

Figure S31: Nine posterior realisations of the underlying genealogy for the analysis of the *Vibrio cholerae* dataset under Kingman's coalescent.

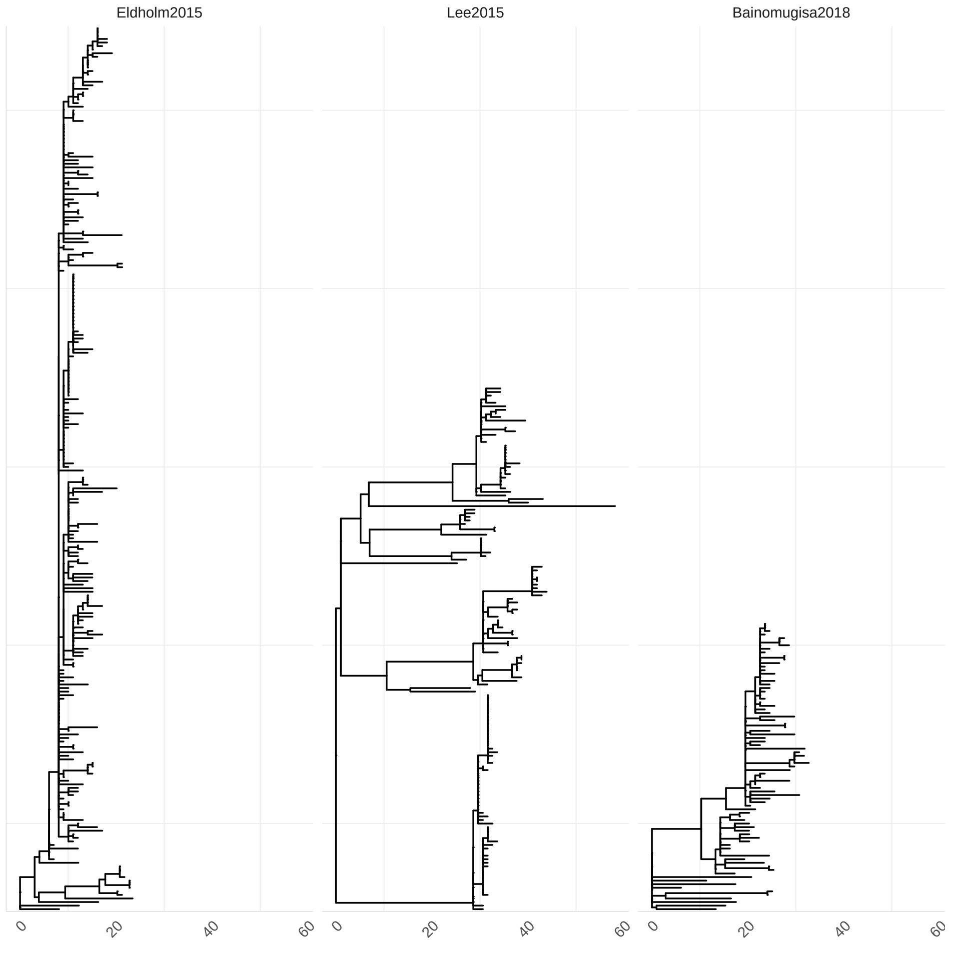

Figure S32: Maximum Likelihood Phylogenies input phylogenies reconstructed using `iqtree -m GTR+G`.

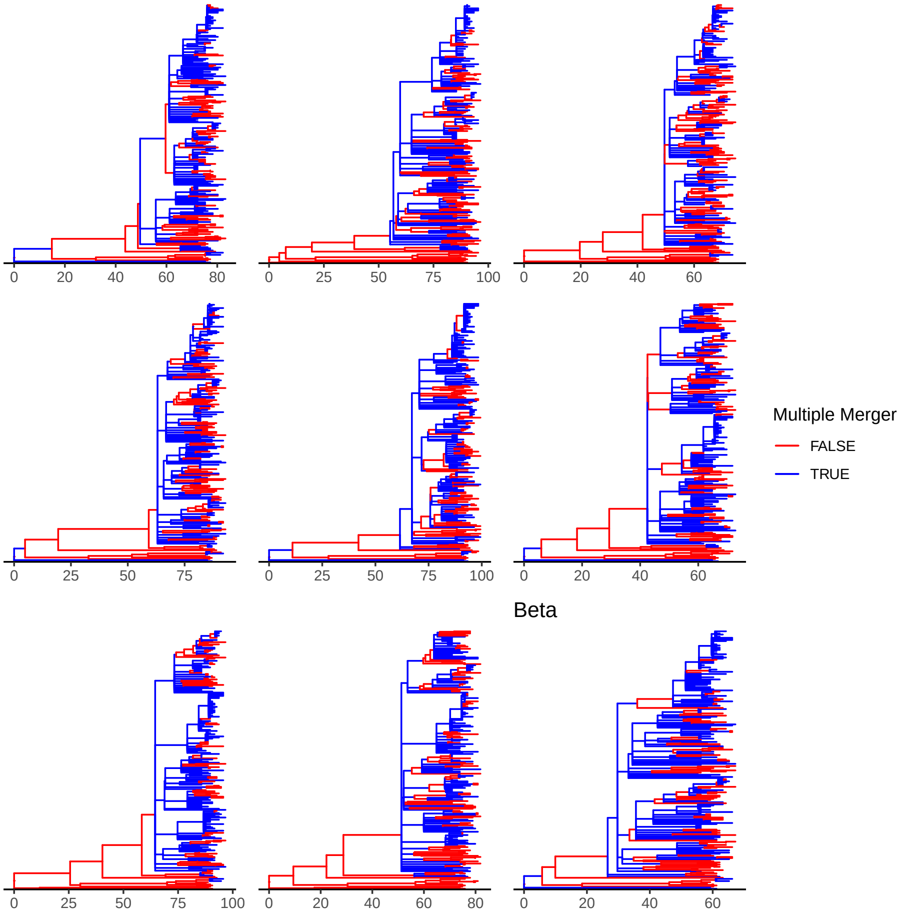

Figure S33: Nine posterior realisations of the underlying genealogy for the analysis of the *Eldholm2015* dataset under the Beta-coalescent.

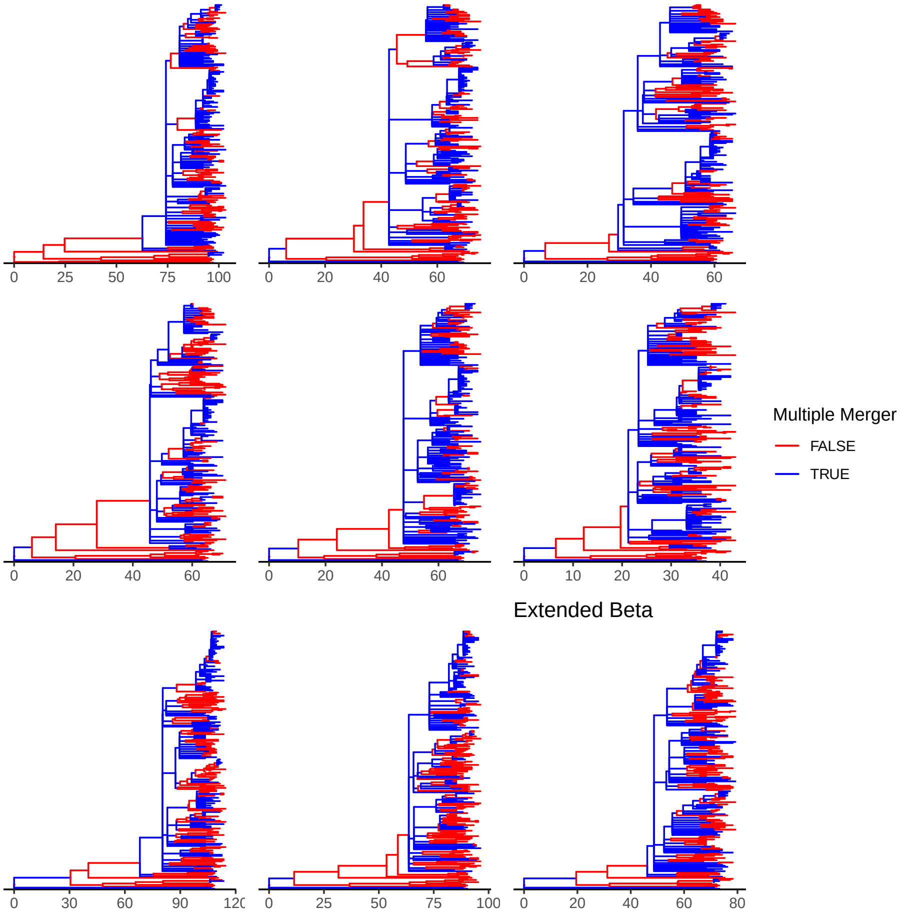

Figure S34: Nine posterior realisations of the underlying genealogy for the analysis of the *Eldholm2015* dataset under the extended Beta-coalescent.

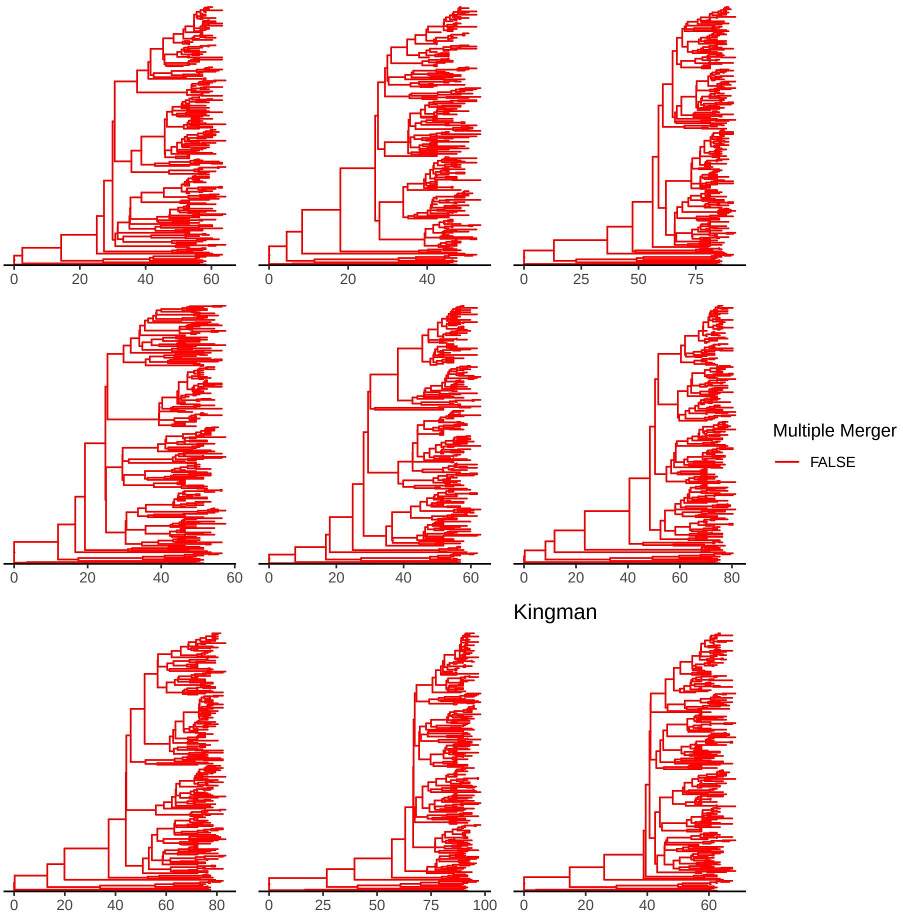

Figure S35: Nine posterior realisations of the underlying genealogy for the analysis of the *Eldholm2015* dataset under Kingman's coalescent.
